# Arbitrary-Shape Dielectric Particles Interacting in the Linearized Poisson–Boltzmann Framework: An Analytical Treatment

**DOI:** 10.1021/acs.jpcb.2c05564

**Published:** 2022-12-06

**Authors:** Sergii V. Siryk, Walter Rocchia

**Affiliations:** CONCEPT Lab, Istituto Italiano di Tecnologia, Via Enrico Melen 83, 16152, Genova, Italy

## Abstract

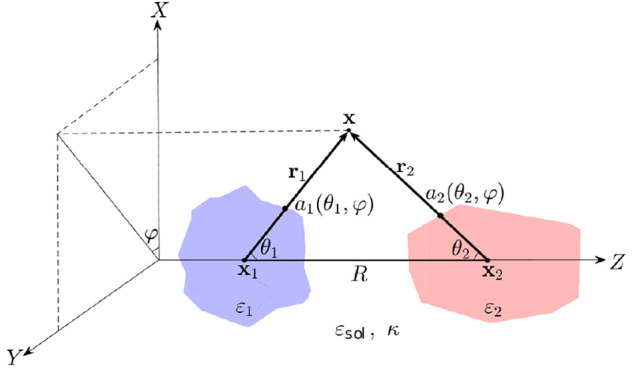

This work considers the interaction of two dielectric particles of arbitrary shape immersed into a solvent containing a dissociated salt and assuming that the linearized Poisson–Boltzmann equation holds. We establish a new general spherical re-expansion result which relies neither on the conventional condition that particle radii are small with respect to the characteristic separating distance between particles nor on any symmetry assumption. This is instrumental in calculating suitable expansion coefficients for the electrostatic potential inside and outside the objects and in constructing small-parameter asymptotic expansions for the potential, the total electrostatic energy, and forces in ascending order of Debye screening. This generalizes a recent result for the case of dielectric spheres to particles of arbitrary shape and builds for the first time a rigorous (exact at the Debye–Hückel level) analytical theory of electrostatic interactions of such particles at arbitrary distances. Numerical tests confirm that the proposed theory may also become especially useful in developing a new class of grid-free, fast, highly scalable solvers.

## 1 Introduction

Electrostatics is pervasive in the physical realm. Especially at the nanoscopic and atomistic scales, it rules plenty of relevant phenomena and plays a crucial role, for instance, in biomolecular systems and biomolecular interactions (e.g., protein–protein association^1^), colloidal solutions, and atmospheric and plasma physics. During biomolecular recognition and binding, electrostatics provides specificity by helping to tune the delicate balance between the desolvation of the binding interfaces and the descreened charged and polar interactions.^1^ At larger distances, the long-range nature of electrostatics allows the interacting partners to spend enough time in close proximity so as to increase the probability of finding the best mutual conformation for binding.^2^

The continuum electrostatics description falls into the category of implicit solvent models.^3^ It represents the system as a low polarizable medium embedded in a highly polarizable one, which may contain dissolved ionic species described as average spatial densities. The Poisson–Boltzmann equation (PBE) and evolutions thereof^4,5^ are continuum electrostatics models. The PBE proved to be very valuable in interpreting many biophysical phenomena. It quite accurately describes the electrostatic interactions occurring between charged particles in solution by combining the electrostatics theory of continuum media with a mean-field approach for the electrostatic potential in the solution.^6^ In this context, the PBE applied to molecular dynamics snapshots was used to characterize porosity and solvent-mediated interactions in nucleosome core particles.^7^

Thanks to its lower computational demand with respect to an atomistic description, the PBE can also be convenient to study supramolecular structures. For example, the electrostatic potential distribution derived via PBE has been used to improve the accuracy of a multiscale generalized Born model, applied to a 40-nucleosome structure,^8^ even though the direct use of a PBE solution via traditional grid-based solvers (such as DelPhi^6,9^ or APBS^10^) becomes impractical for very large systems.

Debye and Hückel (DH) proposed^11^ a continuum method for the estimation of the solvation free energy of spherical ion in 1923. In this model, the energy arises from the electrostatic interaction between the ion and the mean potential generated by the surrounding counterion cloud and by the polarizable solvent medium. In their approach, the Poisson equation is solved for the electrostatic potential Φ inside the spherical ion while in solution the free charge linearly responds to the local potential. This formalism corresponds to the linearization of the PBE in spherical symmetry^11−14^ (let us recall that in general the PBE is a second-order nonlinear elliptic partial differential equation^15^). The linearized PBE (LPBE) (for the above reasons also called the DH equation) is often used for low charged systems^16^ originating sufficiently small potentials Φ (i.e., as  which is equivalent to |Φ| < *k*_B_*T*/*e* = 25.7 mV at a room temperature of 25 °C, where *k*_B_, *e*, and *T* are the Boltzmann’s constant, elementary charge, and absolute temperature, respectively^12,17−21^). This, however, does not diminish the importance of studying the behavior of the LPBE also in the case of highly charged objects: their electrostatics may still be correctly described at sufficiently long distances (as compared to the Debye length) by the usual DH approximation provided that the sources of the electric field are properly renormalized.^22−32^ (See also the recent ref (33) for additional comments concerning the ranges of applicability of the DH theory.) This once again emphasizes the importance of a thorough study of the DH approximations, both theoretically and numerically, and justifies the constant stream of works related to the LPBE (see recent refs (2, 5, and 34−44), and references therein).

Since the initial developments, considerable efforts have been invested into extending DH theory and deriving analytical approximations for the solution of the LPBE in the more complex case of two interacting particles of *spherical* shape. Recently, Filippov and Derbenev and co-workers have published several works^19,20,45^ exploring the electrostatic force between two charged polarizable spheres immersed in an electrolytic solution or in equilibrium plasma. Other relevant studies important to mention here are refs (2, 12, 40, 41, and 46−52). Let us note that the existing literature largely focuses on treating the case of a system of two spherical particles with special symmetries also in the charge (especially, azimuthal symmetry). Notable exceptions to this are ref (47) (aimed at finding simple analytical expressions for the interaction energy by fitting them to a numerical solution of the DH equation in the case of two equal-radii spheres), ref (48), which extends the results of the previous work to two spheres with unequal radii, ref (50) (aimed at treating interactions with the imposed nonuniform surface potentials), ref (51) (estimating the interaction energy for two rigid globular proteins with arbitrary charge distributions at large separations), and ref (53) (electrostatic treatment of two permeable spherical shells). Let us also note the very recent ref (40) that is the first to provide, in November 2020, the general rigorous (exact at the DH level) treatment of many-sphere systems. On the basis of that work, a further assessment of pair- and multisphere effects is made in ref (41). More detailed review of the literature can be found in ref (2).

Albeit the problem of analytically describing the interactions between two conducting spheres is quite long-standing, similar studies on the interactions of two polarizable dielectric spheres (for instance, within the DH framework) arose only relatively recently and are still growing.^19,54−60^ It is now known that polarization can strongly influence the electrostatic interactions between dielectric particles, especially at close interparticle separations, and lead to rather counterintuitive effects [that go beyond the scope of the standard Coulombic/singly screened (DLVO—Derjaguin–Landau–Verwey–Overbeek) interaction terms neglecting polarization], such as the attraction between like-charged particles.^2,19,51,54,55,57,61−64^ These effects were observed mostly numerically, but also experimentally, and especially for dielectric spheres. Some partial and approximate analytical results toward the quantification of higher-order terms, which go beyond the conventionally used Coulombic/singly screened ones, for the potential and interaction energy in a two-sphere system were obtained in refs (12, 46, 51) (doubly screened terms), and ref (40) (triply screened terms), while no general results were known for higher screening orders until the very recent ref (2) to the best of our knowledge. Thus, their study and analytical quantification still remain of great importance. In this respect, in the recent ref (2) the authors presented novel two-center spherical re-expansions that are free of any restrictive symmetry assumptions and improve on the previous developments bypassing the conventional expansions in modified Bessel functions. On the basis of them, they constructed asymptotic expansions in ascending order of Debye screening terms for the electrostatic potential and the total electrostatic energy in the case of two spheres bearing arbitrary charge distributions. This made it possible to explicitly quantify *all* (*k* ≥ 0)-screened terms of the potential coefficients and electrostatic energy and thereby to refine a number of partial approximate results previously reported in the literature (see details in ref (2), section II C) for any two-sphere system. In the same work, it was further demonstrated that even in the (simplest) case of two centrally located point charges the (*k* ≥ 2)-screened terms may significantly exceed the conventional singly screened (*k* = 1) DLVO term.^2,19,41,65,66^ This imbalance can only increase when higher-order multipoles are present or when particles have a large dissimilarity (in size, charge, etc.). This emphasizes the importance of developing a rigorous (exact at least at the DH level) electrostatic analytical theory for *arbitrary-shape* polarizable dielectric particles at arbitrary interparticle separations and without any assumption on charge or system symmetries and particle sizes (as often found also in the recent literature—see the detailed overview in ref (2), section III). It is worth noting that, apart from the extensively discussed case of spherical particles, recently exact (analytical) results were also obtained for interaction between a charged dielectric sphere and a planar surface^67^ and between two dielectric spheroids^21^ in the Poisson limit, i.e., at zero ionic strength. Moreover, recent studies concerned the interaction between cylinders and a flat surface^68,69^ and the numerical evaluation of forces in a cylinder–sphere system^70^ based on the so-called surface element integration approach (proposed in refs (71 and 72)), which attempts to extend the DLVO theory to the interaction of differently shaped particles with a (relatively) flat surface. Here, let us note that the DLVO theory was originally developed for treating spherical particles (colloids), while here it approximates a shape by an “equivalent” spherical diameter.^68,69^ Finally, let us also quote a very recent ref (73), which derives a simple closed-form formula for the apparent surface charge and the electric field generated by a molecular charge distribution in aqueous solution (in the Poisson limit, i.e., at zero ionic strength). However, no rigorous general analytical solution for the case of two polarizable arbitrary-shape dielectric particles is currently known, to the best of our knowledge. By rigorous and general throughout this paper we mean both exact at the DH level and free from any restricting hypothesis on geometry or symmetry of the system. The current paper aims at bridging this gap, namely:

(1) In order to rigorously treat the mutual polarization of arbitrary-shape particles at arbitrary distances we derive a novel spherical re-expansion result for the LPBE solution. In this approach, no restrictive assumptions on either the symmetry of potentials/charge distributions or on the ratio of *r*_*i*_ and *R* (see section 2 below for definitions of all symbols) are imposed. This is an interesting advance with respect to the existing literature—see, for instance, refs (2, 12, 19−21, 40, 41, 49−53, 74−81), which require *r*_*i*_ < *R* (see details in section 3.1 and Appendix A below).

(2) On this basis, we derive relations 11 and 12 for determining the potential expansion coefficients both inside and outside two arbitrary-shape dielectric particles—see section 4.1 for details. These relations do not rely on any restrictive assumption and lead to known expressions, such as those in the recent ref (21), for the particular case of azimuthally symmetric interactions of two dielectric spheroids at zero ionic strength (the proof of this fact, however, requires some rather fine mathematical calculations which are postponed to Appendix F).

(3) These relations allow us to construct small-parameter (∝ e^–*κR*^/*R*, see details in section 4.2) asymptotic expansions for potential coefficients and the corresponding total electrostatic energy in ascending order of Debye screening, hereby generalizing the results of recent ref (2) (see section 4.3).

(4) Finally, we perform a brief numerical benchmarking of our analytical theory against the finite-differences based DelPhi ver. V numerical solver^6,9^ on several model numerical examples (section 5). Unlike conventional grid-based approaches, our methodology requires no external box boundary conditions and computation time is relatively independent of the distance between particles. Importantly, being grid-free, it does not suffer from numerical artifacts associated with the discretization of the equation. Numerical tests show that the calculation time using the theory proposed in this article can be several orders of magnitude smaller than the corresponding calculation times in DelPhi. Interestingly, different contributions to the potential can be calculated separately with ease.

This paper is organized as follows. Section 2 formulates the problem of two interacting dielectric particles relying on the LPBE (DH) model. The transmission conditions treatment and the derivation of novel two-center re-expansion are presented in section 3. Section 4 presents the derivation of relations for determining the potential coefficients and small-parameter expansions. Section 5 demonstrates several numerical tests. Finally, technically subtler derivations, proofs, and auxiliary topics, that are instrumental in (and integral for) this study, are postponed to Appendixes.

## 2 Electrostatic Problem Formulation

**Figure 1:**
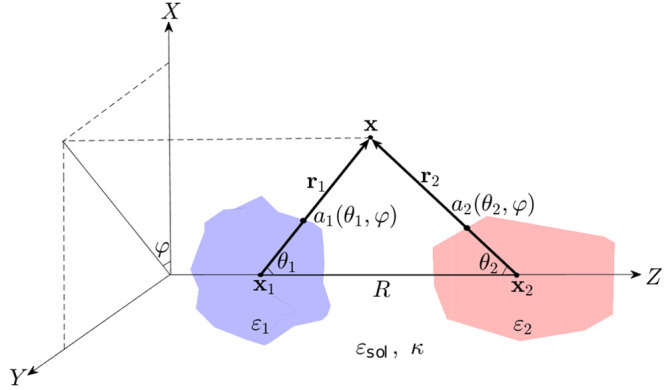
General geometry of the system under consideration.

Let us consider a general system consisting of two nonintersecting dielectric particles *i* and *j*, with dielectric constants ε_*i*_ and ε_*j*_. We adopt two spherical coordinate systems with their origins associated with centers **x**_*i*_ and **x**_*j*_ of the particles (let us note that since the LPBE is a Helmholtz-type equation, it cannot be solved in the standard bispherical coordinate system through separation of variables, see ref (19)). Without loss of generality, one can assume that **x**_*i*_ and **x**_*j*_ lie on the Cartesian axis *Z*, while the axes *X* and *Y* are fixed. The corresponding particle surfaces are then parametrized in these spherical coordinate systems by the radial distances *a*_*i*_ and *a*_*j*_ depending on the (polar and azimuthal) angles, see Figure 1. The particles are separated by a distance *R* between their centers. Without loss of generality we will assume henceforth that  and *j* = 3 – *i*. These particles are in an electrolytic solution (for instance, water, and mobile ions) with dielectric constant ε_sol_ and Debye length κ^–1^.

The electrostatic potential Φ_in,*i*_ inside the *i*th particle  satisfies the Poisson equation^82^
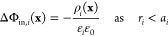
1where *r*_*i*_ is the radial coordinate of  measured from the center **x**_*i*_ of the *i*th particle (so that *r*_*i*_ = ∥**r**_*i*_∥, **r**_*i*_ = **x** – **x**_*i*_), and ρ*_i_*(**x**) denotes the charge density inside the *i*th particle. Simultaneously, consistent with the Debye–Hückel (DH) model, the potential Φ_out,*i*_ in the surrounding medium caused by the presence of the *i*th particle satisfies the LPBE:^12,19−21,46^
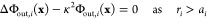
2

Due to the superposition principle the self-consistent total electrostatic potential Φ(**x**) of the whole system is then^12,19−21,46^
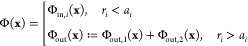
3, subjected to the following boundary conditions on the particles’ surfaces:
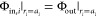
4a
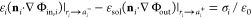
4bwhere **n**_*i*_ is the unit normal vector and σ_*i*_ is a permanent free charge density distribution on the surface *r*_*i*_ = *a*_*i*_ of the *i*th particle (if any); since we are further interested in dielectric systems with no fixed free surface charge we can assume σ_*i*_ = 0 (with no loss of generality of considerations, since formulations with and without fixed surface charge are essentially equivalent from the mathematical point of view, at least for the spheres; see refs (40 and 83)). The notation *r*_*i*_ → *a*_*i*_^±^ here indicates approaching the surface of the particle from the interior (−) or the exterior (*+*) side. Also, *A* ≔ *B* or *B* ≕ *A* denotes that the value of *A* is determined (defined) by the value of *B*.

The general solution of eq 1 can be represented in the form
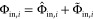
5where Φ̂_in,*i*_ is the given particular solution to 1 that represents the standard Coulombic potential in infinite space for the distribution ρ_*i*_(**x**); in particular, explicit singling out of the Φ̂_in,*i*_ term provides a convenient way to extract the self-energy contributions from the total electrostatic energy of a system (see, e.g., refs (2, 12, 40, 82)). Then, introducing dimensionless radial coordinates *r̃*_*i*_ ≔ *κr*_*i*_ and denoting *ã*_*i*_ ≔ *κa*_*i*_, , eqs 1 and 2 boil down to the following homogeneous equations:
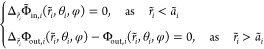
6 and Δ_*r̃*_*i*__ denotes the Laplace operator with *r̃*_*i*_ as the radial spherical coordinate.

Physically feasible general solutions to 6 (such that |Φ̃_in,*i*_| < ∞ as *r̃*_*i*_ → 0^+^ and Φ_out,*i*_ → 0 as *r̃*_*i*_ → + ∞) can be expanded in modified Bessel functions of the second kind, *K*_*n*+1/2_(*r̃*_*i*_) (Macdonald functions)^84^ and associated Legendre polynomials *P*_*n*_^*m*^(*x*) =  (where *P*_*n*_(*x*) is the *n*th standard Legendre polynomial)^85^ in the real-valued form as follows:
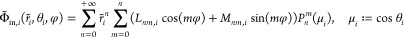
7a
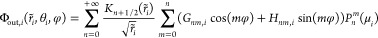
7bwith some real-valued expansion coefficients *L*_*nm*,*i*_, *M*_*nm*,*i*_, *G*_*nm*,*i*_, *H*_*nm*,*i*_ to be determined from boundary conditions 4. Appendix I briefly summarizes the minimal necessary information on the modified Bessel functions used in the text. Let us also note, however, that many authors^40,41,51,78^ prefer to express potentials 7 in terms of complex-valued spherical harmonics *Y*_*nm*_(θ_*i*_, φ) =  instead of using the real-valued ones (that is, cos(*mφ*) *P*_*n*_^*m*^(μ_*i*_), sin(*mφ*) *P*_*n*_^*m*^(μ_*i*_)); this case and the corresponding re-expansion 65 for the DH potential are discussed in Appendix G.

Finally, let us briefly recall that the total electrostatic energy  (within the LPBE framework) is given by^6^
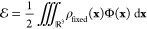
8where ρ_fixed_ is the fixed charge distribution (of any kind, see 1) present in the system. Energy  of a given two-particle configuration (Figure 1) can also be decomposed as^2^
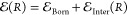
where  is an *R*-independent energy component representing the sum of the (Born) energies of two particles, while  represents the mutual interaction energy of particles at finite *R*.

## 3 Re-Expanding the External Potentials: Theory and Numerics

### 3.1 Treating Boundary Conditions: Deriving Novel Re-expansions in Terms of Associated Legendre Polynomials

**Figure 2:**
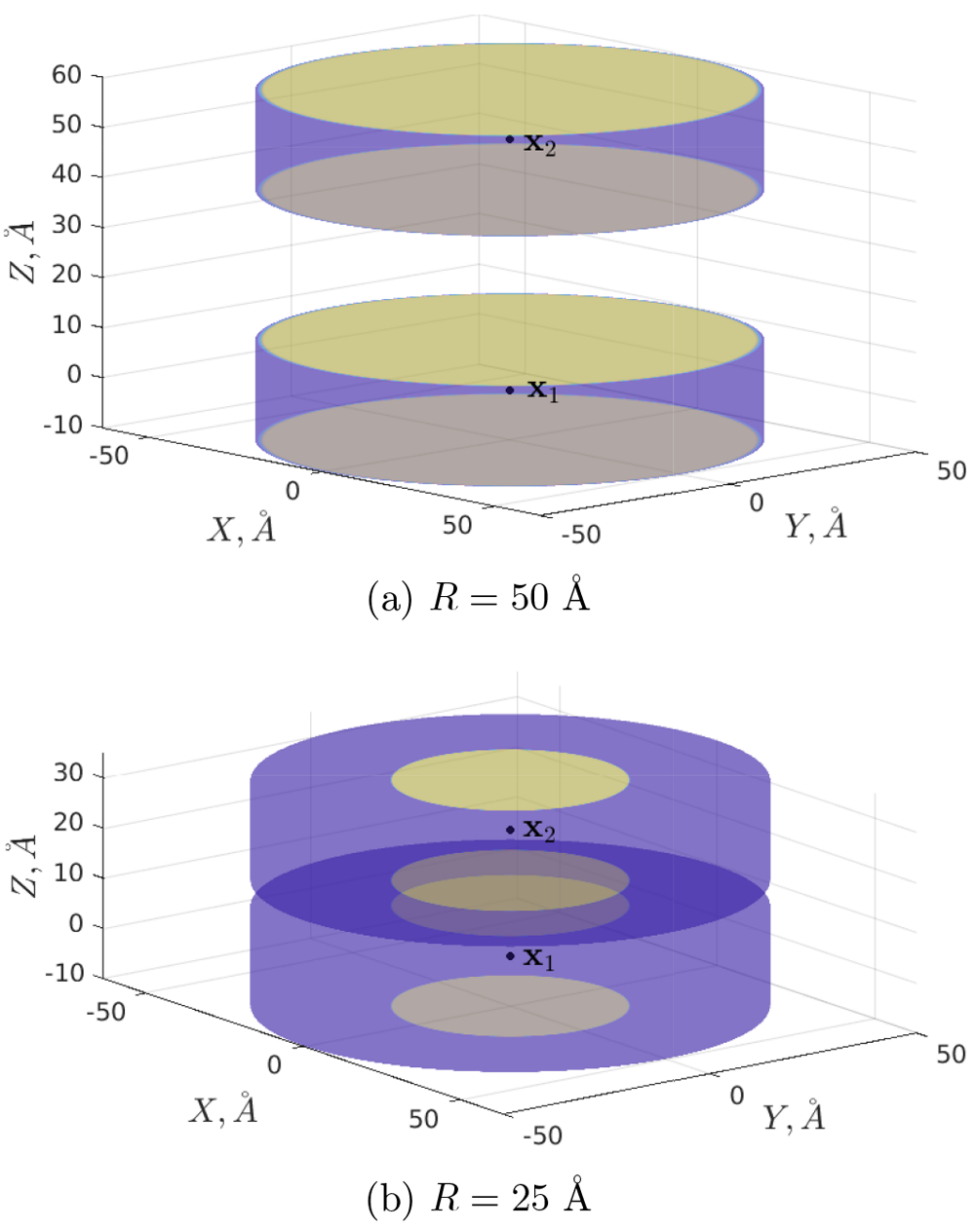
System of two cylinders with a height of 20 Å and a radius of 50 Å centered at **x**_1_ = (0,0,0) and **x**_2_ = (0,0,*R*). All lengths are measured in angstroms (Å). The darker color scale represents surface areas where *r*_*i*_ > *R*.

The main difficulty in determining expansion coefficients in 7a and 7b from the boundary conditions 4 is that the expansions for Φ_out,*i*_(*r̃*_*i*_, θ_*i*_, φ) and Φ_out,*j*_(*r̃*_*j*_, θ_*j*_, φ) refer to different spherical coordinate systems and corresponding spherical harmonics. For instance, in order to impose boundary conditions 4, the authors of recent refs (19−21) propose to re-expand the potential, say Φ_out,*j*_, in terms of coordinates (and corresponding orthogonal Legendre polynomials) of the other sphere *i*; let us note that this is quite a conventional approach which is followed by many authors, see refs (2, 12, 19−21, 40, 41, 49−53, 74−81), allowing one to handle the corresponding boundary conditions correctly from the mathematical point of view. Let us also note that, in contrast to the well-known works in refs (12, 49, and 74), the theory built in refs (19−21) does not make use of the additional reflection symmetry about the plane bisecting the line connecting the spheres’ centers and the corresponding equality of the expansion coefficients of Φ_out,*i*_ and Φ_out,*j*_, which rely on the assumption that the radii of the spheres are equal. Thus, the expansion of Φ_out_ built in refs (19−21) is in principle applicable to the case of spheres with different radii. However, the theory and re-expansions presented in refs (19−21) assume the azimuthal symmetry for the potentials 7a and 7b (i.e., independence of φ) and therefore would not be able, e.g., to deal with an arbitrary orientation of the free dipoles located inside the dielectric spheres. Here, we intend to fill this gap of refs (19−21) and to expand upon the corresponding re-expansions to include general cases devoid of any angular symmetry. Another important feature of the re-expansion presented here is that it does not impose the restrictive inequality *r*_*i*_ < *R*, in contrast to re-expansions derived in the existing literature.^2,12,19−21,40,41,49−53,74−81^ This allows us to consider the case of very close arrangement of arbitrary highly irregular dielectric particles. As an example, Figure 2 illustrates the simplest example of such a situation when two flat thick circular dielectric disks are located very close to each other (let us note that despite the considerable interest to the interactions between two flat membranes/disks^14,18,86,87^ we are not aware of any complete, DH-exact, analytical description of this kind of system).

Namely, to this end, we advance the following representation (re-expansion) of the potential Φ_out,*j*_:
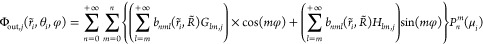
9where the re-expansion coefficients *b*_*nml*_ are determined via 29 and *R̃* ≔ *κR*, , *j* = 3 – *i*. The quite technical derivation of 9 is given in Appendix A.

Let us emphasize that 9 provides an expansion of the potential Φ_out,*j*_ (originally referred to coordinates of the *j*th spherical system and having harmonic expansion coefficients *G*_*lm*,*j*_, *H*_*lm*,*j*_) through harmonics and coordinates referenced now to the *i*th system. Analytical properties (alongside some important particular cases) of the re-expansion coefficients *b*_*nml*_ are described in Appendices A and B.

### 3.2 Numerical Calculation of Potentials in Practice: Truncating the Re-expansions and Approximating the Re-expansion Coefficients

Since the quantities (∑_*l* = *m*_^+∞^*b*_*nml*_(*r̃*_*i*_, *R̃*)*G*_*lm*,*j*_) and (∑_*l* = *m*_^+∞^*b*_*nml*_(*r̃*_*i*_, *R̃*)*H*_*lm*,*j*_) in 9 as well as the re-expansion coefficients *b*_*nml*_(*r̃*, *R̃*) of 29 in general contain infinite sums, in practical calculations, to determine the potential expansion coefficients *L*_*nm*,*i*_, *M*_*nm*,*i*_, *G*_*nm*,*i*_, *H*_*nm*,*i*_,  one needs to apply a truncation to a finite number of terms. This is usually done according to the required accuracy, which is often estimated by tracking the evolution of some key quantity, e.g., the electrostatic energy  of the system as done in the convergence estimate for grid-based solvers.^6,9^ Interestingly, only the energy components depending on potential coefficients subjected to further changes need to be recalculated; see 7a and 8. To this end, we propose and then numerically benchmark (section 5) the approximation methodology, the simpler “azimuthally symmetric” version of which for *m* = 0 was proposed in ref (19) and successfully verified to be effective in refs (20 and 21). The approximation methodology we propose consists of the following two points: (1) only coefficients *b*_*nml*_(*r̃*, *R̃*) with *n* + *l* – *m* ≤ *n*_max_ are to be calculated, while all of the others are assumed to be zero; (2) further additional constraints *s* ≤ *n*_max_ – *l* and *k* ≤ *n*_max_ + *m* – *l* – *s* are enforced on the infinite series 29, where *n*_max_ ≥ *m*, is a given fixed user-defined threshold. For *m* = 0, the proposed approximation methodology simply boils down to that of refs (19 and 20). Simple algebraic calculations indicate that this approximation methodology calculates the exact values of the coefficients *b*_*nmm*_(*r̃*, *R̃*) (see 36) if *n*_max_ ≥ *n* is taken; however, this is not the case, e.g., for the coefficient *b*_*nml*_(*r̃*, *R̃*) with general triplet (*n*, *m*, *l*) of indices (an illustrative example of the convergence of the re-expansion coefficients, approximated by the methodology just described, is given in Appendix B.3).

## 4 Expansion Coefficients for the Potentials, Small-Parameter Expansions: Theory

### 4.1 Derivation of Relations Governing the Potential Coefficients

Determining the unknown potential expansion coefficients *L*_*nm*,*i*_, *M*_*nm*,*i*_, *G*_*nm*,*i*_, *H*_*nm*,*i*_ of 7 completely solves the problem of finding the electrostatic potential.

With using 5, 7, and 9, after algebraic transformations boundary condition 4a acquires the following expanded form:
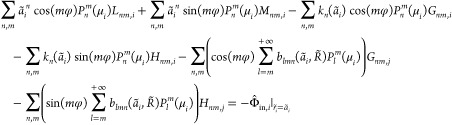
where, to shorten the recording of formulas, it is denoted that *k*_*n*_(*x*) ≔ *K*_*n*+1/2_(*x*)/, *ã*_*i*_ = *ã*_*i*_(θ_*i*_, φ) (with 0 ≤ θ_*i*_ ≤ π, 0 ≤ φ < 2π so that the entire surface of particle *i* is covered), , *j* = 3 – *i*, and  denotes the double sum (likewise to 7) over indices (*n*, *m*) with *n* ≥ *m* ≥ 0 or *n* ≥ *m* ≥ 1 for the expressions involving coefficients *L*_*nm*_, *G*_*nm*_, or *M*_*nm*_, *H*_*nm*_, respectively. Then, multiplying both sides of this equality by
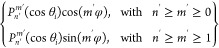
10and integrating over Ω_*i*_ ≔ {(θ_*i*_, φ)|0 ≤ θ_*i*_ ≤ π, 0 ≤ φ < 2π} (i.e., over the entire surface of particle *i*) with weight sin θ_*i*_, one gets the following linear systems with respect to the unknown coefficients of 7:
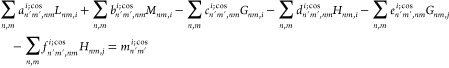
11a
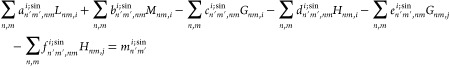
11bwhere coefficients *a*_*n*′*m*′,*nm*_^*i*; cos^, *b*_*n*′*m*′,*nm*_^*i*; cos^, *c*_*n*′*m*′,*nm*_^*i*; cos^, *d*_*n*′*m*′,*nm*_^*i*; cos^, *e*_*n*′*m*′,*nm*_^*i*; cos^, *f*_*n*′*m*′,*nm*_^*i*; cos^, and *m*_*n*′*m*′_^*i*; cos^ are given by 48 (due to their cumbersomeness, the corresponding expressions are all placed in Appendix D). The values of *a*_*n*′*m*′,*nm*_^*i*; sin^, *b*_*n*′*m*′,*nm*_^*i*; sin^, *c*_*n*′*m*′,*nm*_^*i*; sin^, *d*_*n*′*m*′,*nm*_^*i*; sin^, *e*_*n*′*m*′,*nm*_^*i*; sin^, *f*_*n*′*m*′,*nm*_^*i*; sin^ and *m*_*n*′*m*′_^*i*; sin^ are defined in the same way, except that the integrals 48 use sin(*m*′φ) instead of cos(*m*′φ) in their integrands. Let us note that in the (simplest) case of a spherical surface (i.e., if *a*_*i*_(θ_*i*_, φ) = constant independent of angles θ_*i*_, φ), functions 10 constitute a complete orthogonal set on a sphere parametrized by Ω_*i*_ so that the integral ∬_Ω_*i*__ (·) sin θ_*i*_ d*θ*_*i*_ d*φ* of the product of arbitrary two such functions with indices (*n*′,*m*′) and (*n*″,*m*″) is zero if (*n*′,*m*′) ≠ (*n*″,*m*″) (in particular, complex-valued spherical harmonics *Y*_*nm*_(θ,φ) are constructed from this basis and fulfill the same orthogonality relation^82^); see 50 below. Then, systems 11 boil down to the identities just resulting from simple collecting/equating the expansion coefficients (of all the functions involved in 4a) at Fourier spherical harmonics sin(*mφ*)*P*_*n*_^*m*^(μ_*i*_), cos(*mφ*)*P*_*n*_^*m*^(μ_*i*_) of the same orders—see, e.g., relation 22 below. This special case is discussed in more detail later in section 4.3. In the general case, when *a*_*i*_(θ_*i*_, φ) does not describe a sphere, the above-described approach of treating boundary conditions essentially follows the idea of spectral Galerkin residual orthogonalization procedure,^88−91^ with the trial and test functions spaces being spanned by set 10.

Let us account for the second boundary condition, that is, relation 4b. Following the same approach as in the previous case of boundary condition 4a and using expressions 46 and 47 to treat differential operators **n**_*i*_ · ∇, and relations  =  (see 69) and  =  =  (see ref (92), eq 8.731), we arrive at the following linear systems with respect to the unknown coefficients of 7:
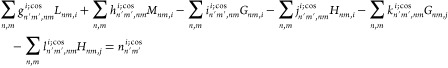
12a
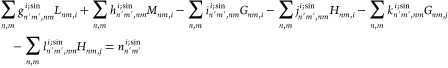
12bwhere coefficients *g*_*n*′*m*′,*nm*_^*i*; cos^, *h*_*n*′*m*′,*nm*_^*i*; cos^, *i*_*n*′*m*′,*nm*_^*i*; cos^, *j*_*n*′*m*′,*nm*_^*i*; cos^, *k*_*n*′*m*′,*nm*_^*i*; cos^, *l*_*n*′*m*′,*nm*_^*i*; cos^, and *n*_*n*′*m*′_^*i*; cos^ are given by 49. The values of *g*_*n*′*m*′,*nm*_^*i*; sin^, *h*_*n*′*m*′,*nm*_^*i*; sin^, *i*_*n*′*m*′,*nm*_^*i*; sin^, *j*_*n*′*m*′,*nm*_^*i*; sin^, *k*_*n*′*m*′,*nm*_^*i*; sin^, *l*_*n*′*m*′,*nm*_^*i*; sin^ and *n*_*n*′*m*′_^*i*; sin^ are defined in the same way, except that the integrals 49 use sin(*m*′φ) instead of cos(*m*′φ) in their integrands.

General systems 11 and 12 read in matrix form as follows:
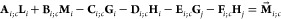
13a
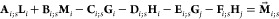
13b
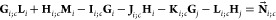
13c
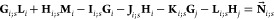
13dwhere evidently , *j* = 3 – *i*, vectors **L**_*i*_ ≔ {*L*_*nm*,*i*_}_0≤*m*≤*n*_, **G**_*i*_ ≔ {*G*_*nm*,*i*_}_0≤*m*≤*n*_, **M**_*i*_ ≔ {*M*_*nm*,*i*_}_1≤*m*≤*n*_, **H**_*i*_ ≔ {*H*_*nm*,*i*_}_1≤*m*≤*n*_, matrices **A**_*i*;*c*_ ≔ {*a*_*n*′*m*′,*nm*_^*i*; cos^}, **B**_*i*;*c*_ ≔ {*b*_*n*′*m*′,*nm*_^*i*; cos^}, **C**_*i*;*c*_ ≔ {*c*_*n*′*m*′,*nm*_^*i*; cos^}, **D**_*i*;*c*_ ≔ {*d*_*n*′*m*′,*nm*_^*i*; cos^}, **E**_*i*;*c*_ ≔ {*e*_*n*′*m*′,*nm*_^*i*; cos^}, **F**_*i*;*c*_ ≔ {*f*_*n*′*m*′,*nm*_^*i*; cos^}, **G**_*i*;*c*_ ≔ {*g*_*n*′*m*′,*nm*_^*i*; cos^}, **H**_*i*;*c*_ ≔ {*h*_*n*′*m*′,*nm*_^*i*; cos^}, **I**_*i*;*c*_ ≔ {*i*_*n*′*m*′,*nm*_^*i*; cos^}, **J**_*i*;*c*_ ≔ {*j*_*n*′*m*′,*nm*_^*i*; cos^}, **K**_*i*;*c*_ ≔ {*k*_*n*′*m*′,*nm*_^*i*; cos^}, **L**_*i*;*c*_ ≔ {*l*_*n*′*m*′,*nm*_^*i*; cos^}, vectors **M⃗**_*i*;*c*_ ≔ {*m*_*n*′*m*′_^*i*; cos^}, **N⃗**_*i*;*c*_ ≔ {*n*_*n*′*m*′_^*i*; cos^}, and corresponding matrices/vectors with subscript *i*; *s* are defined by coefficients with superscript *i*; sin in exactly the same way. Let us also note that, by construction, indices (*n*′,*m*′) enumerate the rows in (sub)matrices 13, while (*n*,*m*) enumerate their columns so that (*n*′,*m*′) run 0 ≤ *m*′ ≤ *n*′ in matrices/vectors with subscript *i*; *c* and 1 ≤ *m*′ ≤ *n*′ in those with subscript *i*; *s*, while (*n*,*m*) run 0 ≤ *m* ≤ *n* in matrices **A**, **C**, **E**, **G**, **I**, **K**, and 1 ≤ *m* ≤ *n* in **B**, **D**, **F**, **H**, **J**, **L**; in addition, when using approximation approach of section 3.2, the corresponding indices *n*′ and *n* are bounded from above by *n*_max_. Next, mutually swapping indices *i* and *j* in 13 one also gets the similar four-equation system but with *i* and *j* interchanged. Thus, combining 13 and the corresponding system with *i* and *j* interchanged, we assemble the following global linear system with the block matrix composed of separate matrix blocks (submatrices) and the (global) unknown column-vector composed of separate column-vectors **L**_*i*_, **M**_*i*_, **G**_*i*_, **H**_*i*_, :
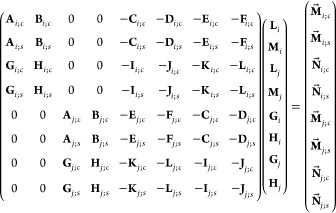
14(there 0 denotes a matrix with all-zero entries). Solving the global linear system 14 one finds **L**_*i*_, **M**_*i*_, **G**_*i*_, **H**_*i*_, . An alternative way for determining these coefficients (yielding explicit expressions for them as well as also allowing us to construct the corresponding small-parameter expansions—see the next section 4.2) is discussed in Appendix E.

Let us note that in the case of a configuration with only one (say, *i*th) dielectric particle present, system 14 simplifies to
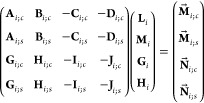
15

### 4.2 Small-Parameter Asymptotic Expansions for Potential Coefficients and Electrostatic Energy in Ascending Order of Debye Screening

The way of deriving explicit solutions to system 14, described in Appendix E, makes it possible to construct small-parameter (∝ e^–*κR*^/*R* as *R* grows unboundedly) asymptotic expansions for the potential coefficients and hence the total electrostatic energy  in ascending order of Debye screening (similar to those of ref (2) built for the case of two spheres):
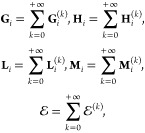
16, where addends with superscript (*k*) are *k*-screened, that is, tend to zero as (e^–*κR*^/*R*)^*k*^ as *R* → + ∞ (or, to be mathematically tidier, they are of order *O*((e^–*κR*^/*R*)^*k*^) as *R* → + ∞).

Indeed, for , matrices **A**_*i*;*c*_, **A**_*i*;*s*_, **B**_*i*;*c*_, **B**_*i*;*s*_, **C**_*i*;*c*_, **C**_*i*;*s*_, **D**_*i*;*c*_, **D**_*i*;*s*_, **G**_*i*;*c*_, **G**_*i*;*s*_, **H**_*i*;*c*_, **H**_*i*;*s*_, **I**_*i*;*c*_, **I**_*i*;*s*_, **J**_*i*;*c*_, **J**_*i*;*s*_ and vectors **M⃗**_*i*;*c*_, **M⃗**_*i*;*s*_, **N⃗**_*i*;*c*_, **N⃗**_*i*;*s*_ in 13 and, hence, , , , , ,  in 54 and , , , , ,  in 55 are *R*-independent.

At the same time, for , matrices **E**_*i*;*c*_, **E**_*i*;*s*_, **F**_*i*;*c*_, **F**_*i*;*s*_, **K**_*i*;*c*_, **K**_*i*;*s*_, **L**_*i*;*c*_, **L**_*i*;*s*_ in 13 and, hence, , , ,  in 54 and , , ,  in 55 are of infinitesimal order *O*(e^–*κR*^/*R*) as *R* grows—indeed, it is easy to assert from 28 and 29 and 68a that e^–*κ R*^/*R* is a common multiplier factor for any element of these matrices. Then, employing the (absolutely convergent) Neumann matrix series  in 58, where *I* is the identity matrix and an *O* ((e^–*κR*^/*R*)^2^)-matrix *N* is either  or , we end up with the following expansions of ,  and  (see 57) in ascending order of Debye screening, :
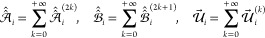
17where addends with superscript (*k*) are *k*-screened. Note also that expansions for  and  contain only even-screened and odd-screened addends, respectively; in particular, doing so one can obtain that
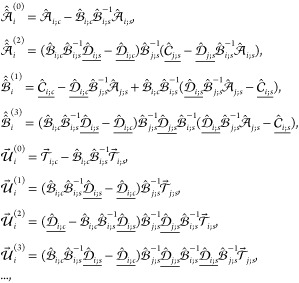
where *O*(e^–*κR*^/*R*)-terms have been underlined (just for better traceability the screening origins of the corresponding left-hand sides). Thence, using Neumann matrix series and employing 17 in 59 one can finally get the desired addends of expansion 16 for **G**_*i*_ ranging in ascending order of Debye screening; in particular, by doing this one can write down the first few addends:
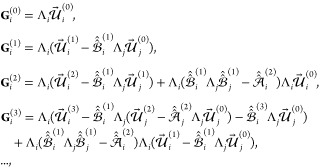
18where it is denoted that , , *j* = 3 – *i*. In turn, the just determined addends {**G**_*i*_^(*k*)^} of expansion 16 of **G**_*i*_ further allow us to construct expansions for the remaining vectors **H**_*i*_, **L**_*i*_, and **M**_*i*_. Indeed, employing Neumann matrix series and identity 56 one has
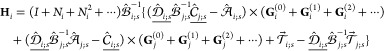
with , or, by collecting terms of the same order of screening, we conclude that
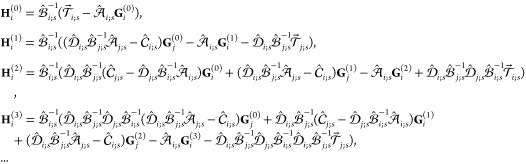
19Then, using 54 one finally obtains
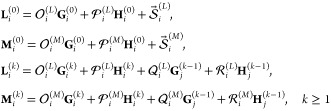
20In turn, employing the coefficients 20 in 7a and further using 8, 5, and 3 immediately yield^2^ the *k*-screened components  of asymptotic expansion (16) for energy .

### 4.3 The Particular Case of Spherical Surfaces

Let us briefly instantiate the analytical theory built in section 4.2 to the case of two spheres and check that it indeed boils down to that of ref (2). Let us first note that in this case it is always possible^82^ to expand Φ̂_in,*i*_(*r̃*_*i*_, θ_*i*_, φ) in multipoles:
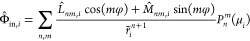
21with some numerical multipolar expansion coefficients {*L̂*_*nm,i*_}_0≤*m*≤*n*_ and {*M̂*_*nm,i*_}_1≤*m*≤*n*_. The integrals 48 and 49 boil down to the simpler expressions 50 (see Appendix D.1); for instance, 11a then simply results in
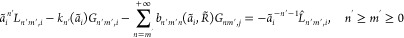
22which coincides with ref (2), eq 43; note that integrals 48 containing sin(*mφ*) completely nullify in 50, for instance, *b*_*n*′*m*′,*nm*_^*i*; cos^, *d*_*n*′*m*′,*nm*_^*i*; cos^ and *f*_*n*′*m*′,*nm*_^*i*; cos^ involved in 11a, whereas only those containing cos(*mφ*) with *m* = *m*′ survive. Using 50 in 54 we further arrive at 51, employing which in 55 we obtain 52. Matrix elements 52, in turn, lead to 53 which coincides with ref (2), eq 45, governing the external potential coefficients in the problem of interaction of two spheres. Furthermore, using 50, 51, and 52 after some algebraic transformations it can be shown that asymptotic expansions addends 18, 19 and 20 for the potential coefficients boil down to those of ref (2), eqs 60 and 69, for the case of a two-sphere system (which in turn were also extensively tested numerically for convergence behavior^2^ and were shown to generalize a number of approximate results previously reported in the literature, see ref (2), sections V and VI), e.g., in the simplest case of two centrally located point charges *q*_*i*_ and *q*_*j*_ (thus, , ) relations 18, 19, and 20 yield *G*_00,*i*_^(0)^ = , *G*_00,*i*_^(1)^ = , *L*_00,*i*_^(0)^ = , *L*_00,*i*_^(1)^ = , from which the known relations (see ref (2), section V)  =  +  (Born energy) and  =  immediately follow.^2^ In the particular case of equally sized spheres *a*_*i*_ = *a*_*j*_ = *a* the latter relation reduces to the well-known DLVO interaction energy^18,63^; see ref (2) concerning the further details on the full analytical solution to the problem of two interacting spheres bearing arbitrary multipoles. Thus, the rigorous (exact at the DH level) electrostatic theory and the corresponding asymptotic small-parameter expansions built in section 4.2 indeed naturally generalize those of ref (2) for the two-sphere system to a much more complicated case of two arbitrary-shape interacting particles.

## 5 Numerical Modeling and Results

In this section we present the numerical illustration of the developed theory; we also compare the corresponding numerical results with those provided by the DelPhi LPBE solver—it is especially well-suited for our current purposes since the version of DelPhi introduced in ref (9) has built-in support for the insertion of the simple geometric objects (e.g., dielectric particles in the form of spheres, cylinders, cones, etc.).

Let us note that the advantage of the approach proposed in the current paper is the relatively small size of the linear system to be solved for calculating the electrostatic potentials in contrast to conventional grid-based methods (where variables/degrees of freedom of the corresponding linear system are usually associated with grid points, and accurate solution of the problem inevitably entails the usage of fine grids), e.g., the matrix of 14 is *N* × *N* with *N* = 4(*n*_max_ + 1)^2^ and, as we will observe from the numerical experiments of the current section, relatively small *n*_max_’s are usually sufficient to produce quite accurate solutions.

Typical values used in the calculations are ε_*i*_ = ε_*j*_ = 2, , and κ^–1^ = 8.071 Å (these solvent parameters are quite typical for systems of biophysical interest, representing an aqueous solution with 0.145 M physiological NaCl concentration at a room temperature of 25 °C). The DelPhi’s settings^6,9^perfil (the percentage of immersion of the physical system into the computational cubic box, to be minimized in order to make the approximation in the external boundary conditions more acceptable) and scale (the reciprocal of one grid spacing, grids/angstrom, to be maximized in order to obtain a finer mesh resolution), which directly affect the time and accuracy of numerical calculations, are described below. Finally, as a stopping criterion in DelPhi we set the value of parameter maxc (grid potential maximum change threshold, in *k*_B_*T*/*e* units, where *k*_B_, *e*, and *T* are Boltzmann’s constant, elementary charge, and absolute temperature, respectively) to 10^–7^ (unless explicitly stated otherwise).

The numerical examples supporting the proposed approach were benchmarked on a PC with an Intel Core i7-9850H CPU @ 2.60 GHz × 12, 15.4 Gb RAM, MATLAB R2021b/R2022a (PC 1); additional tests (see Figure 5) were performed on an Intel Xeon(R) W-2265 CPU @ 3.50 GHz × 24, 125.5 Gb RAM, MATLAB R2022a (PC 2). DelPhi was run on cluster with Intel Xeon(R) v3 CPU @ 2.30 GHz × 40, 503.8 Gb RAM.

Numerical experiments are discussed in the next section, 5.1. In addition, in section 5.2 we also propose and discuss a simple approach, based on the Tikhonov regularization theory, to improve the accuracy and stability of potential/energy calculations at high *n*_max_.

### 5.1 Numerical Experiments Results

#### 5.1.1 Interacting Cones

**Figure 3:**
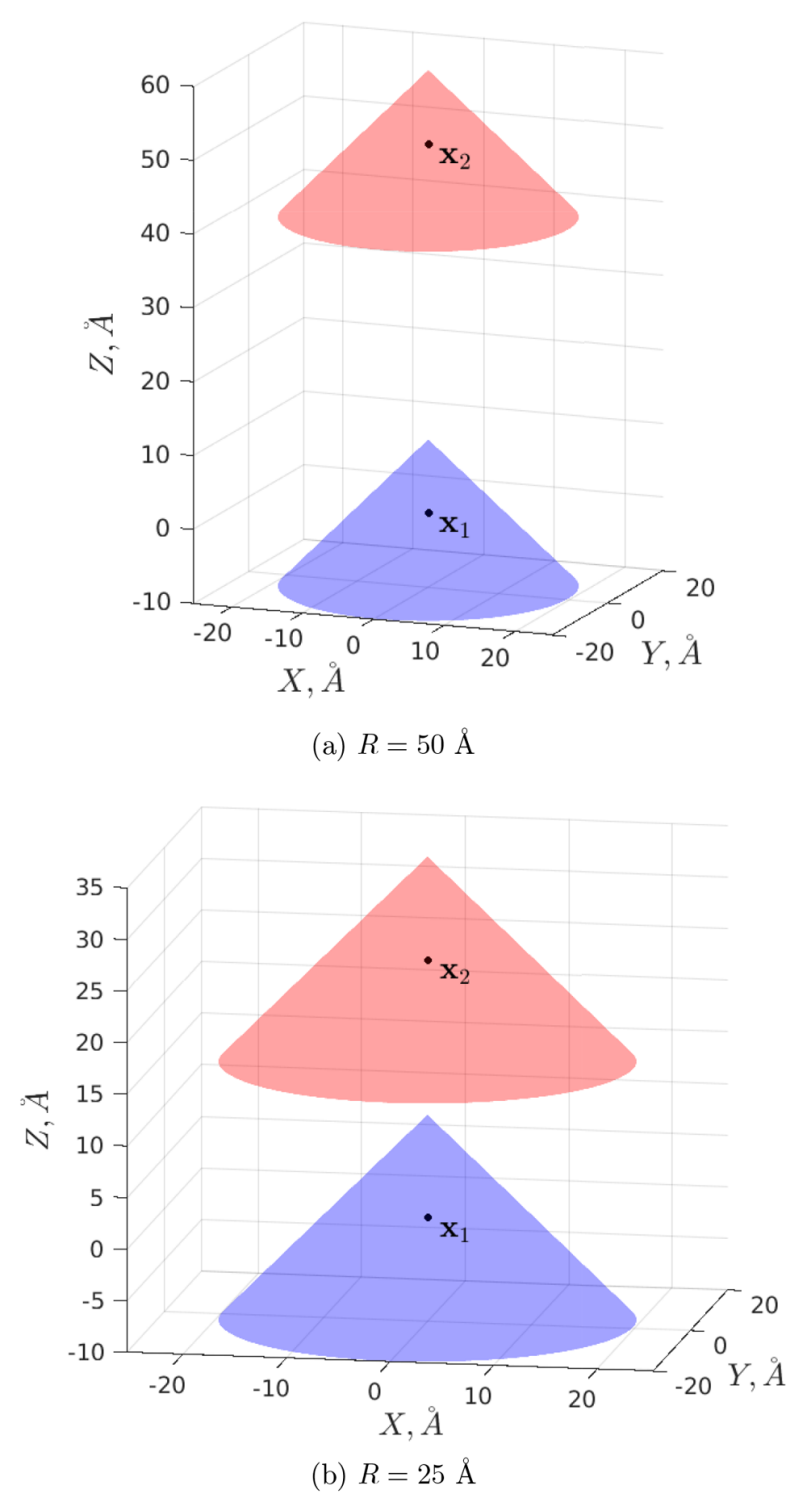
System of two cones centered at **x**_1_ = (0,0,0) and **x**_2_ = (0,0,*R*). All lengths are measured in angstroms (Å).

Let us now consider a system of two equal cones with opening angle of π/2:
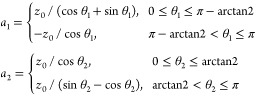
23where 2*z*_0_ is the cone height, *z*_0_ = 10 Å, and two point charges *q*_1_ = *q*_2_ = 10*e* are situated in their centers **x**_1_, **x**_2_. The corresponding graphical representations for the distances *R* = 50 Å and *R* = 25 Å are shown in Figure 3. These cases are azimuthally symmetric (thus, potential coefficients with *m* > 0 nullify and therefore do not need to be calculated, in general).

**Figure 4:**
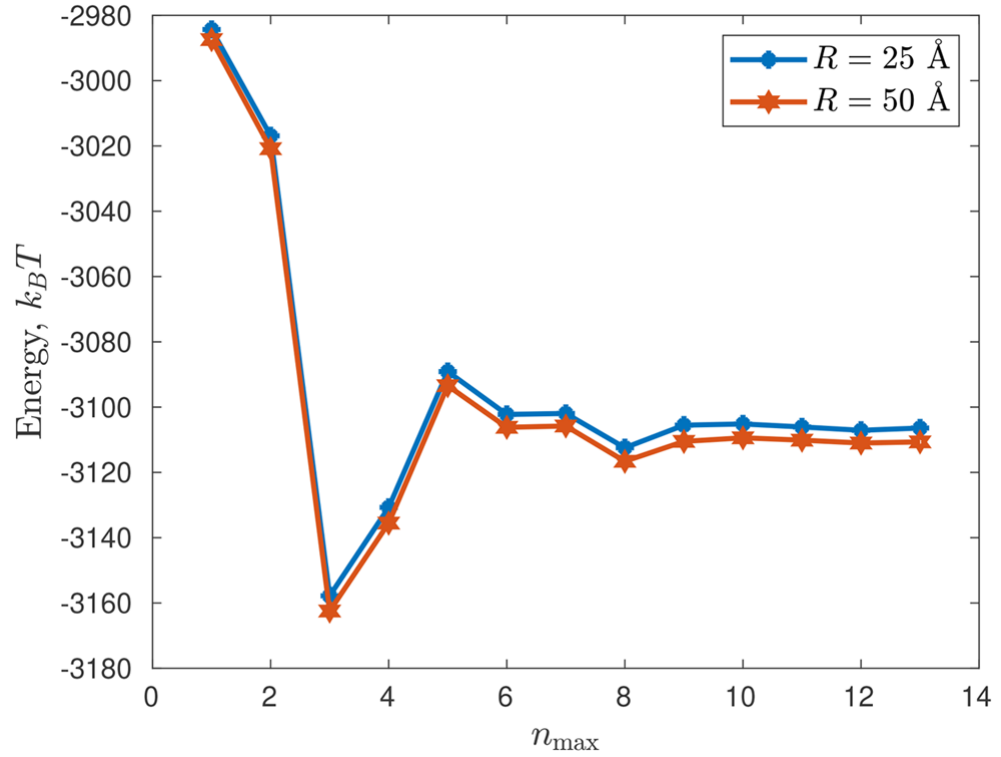
Total electrostatic energy  at different *n*_max_’s.

**Figure 5:**
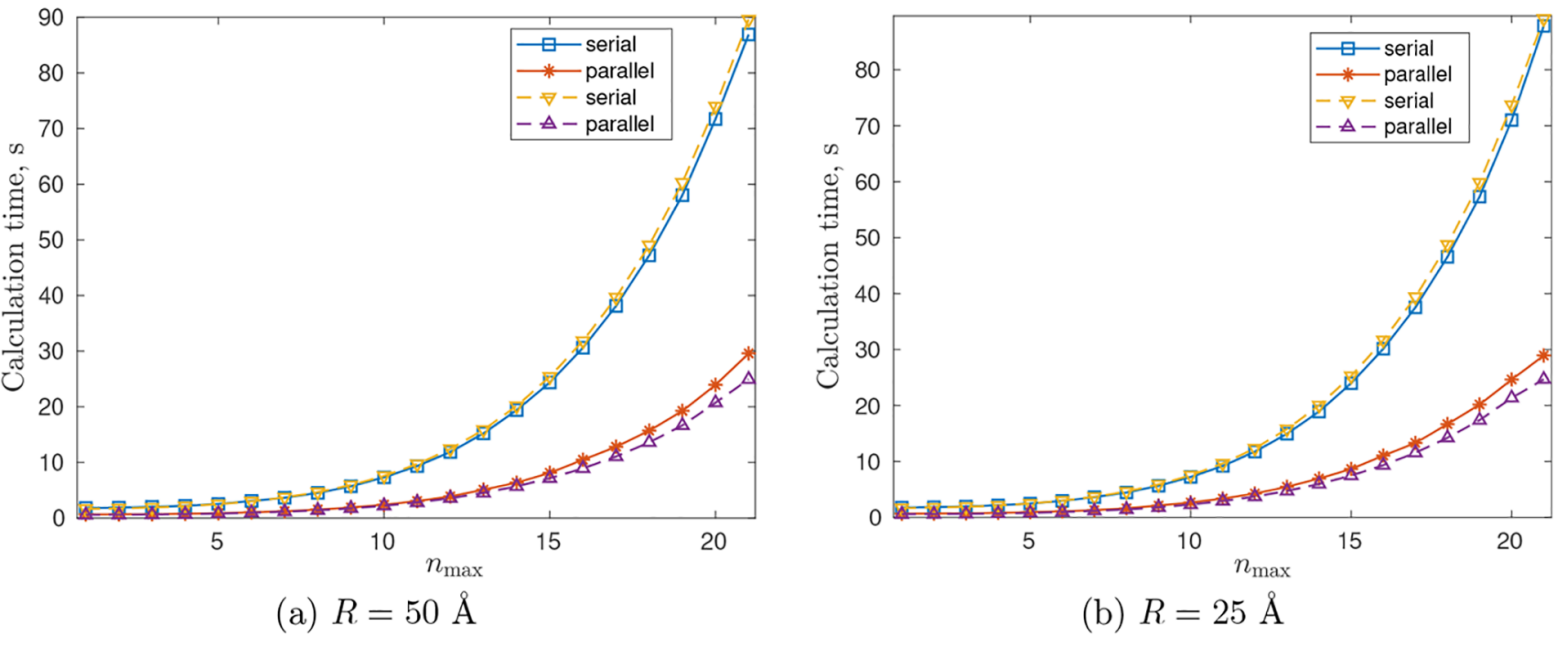
Computation time at different *n*_max_’s. The curves labeled “serial” imply a fully sequential calculation, while “parallel” curves show the calculation times in the case where the calculation of integrals 48 and 49 is parallelized between four parallel workers so that different parts of a surface are processed by different workers (in the example under consideration, two workers per cone: one for processing the side surface of the cone and another for processing the cutting plane). Dashed curves correspond to PC 2.

Then, Figure 4 demonstrates the total electrostatic energy  for *R* = 25 Å and *R* = 50 Å at different *n*_max_’s calculated by the proposed methodology; the figure shows that  stabilizes rather quickly and practically stops changing after *n*_max_ = 9. Table 1 reports in more detail the numerical values of  at *R* = 50 Å for some *n*_max_’s together with the corresponding calculation timings in serial and parallel (values in parentheses) modes. Table 1 clearly demonstrates that the calculation time using the theory proposed in this article can be several orders of magnitude less than the corresponding calculation times in DelPhi (see Figure 5 for more details on the calculation times using the proposed theory in the current example, in both cases of serial and parallel computations). Figure 5 shows that the computation time in our approach has little dependence on *R* (moreover, since only the matrices **E**_*i*;*c*_, **E**_*i*;*s*_, **F**_*i*;*c*_, **F**_*i*;*s*_, **K**_*i*;*c*_, **K**_*i*;*s*_, **L**_*i*;*c*_, and **L**_*i*;*s*_ depend on *R*, and this dependence is of the order of *O*(e^–*κR*^/*R*), which causes these matrices to decay rapidly, see section 4.2, then in practice, the integrals 48 and 49 may be calculated even faster for larger *R* within a given accuracy). In contrast to this, grid-based approaches must cover the whole system under interest by a computational domain; thus, decreasing *R* decreases the grid size too and, respectively, the total time of calculations, e.g., for *R* = 25 Å the corresponding DelPhi calculation times (for the same scale and perfil as consecutively listed in Table 1) are 553.83, 9936.03, 14 868.76, and 46 015.28 s, with the corresponding gridsize equal to 451^3^, 937^3^, 1051^3^ and 1351^3^, respectively.

**Table 1:** Energy Calculations, *R* = 50 Å^a^

energy [*k*_B_*T*]	time [s]
DelPhi (scale = 3, perfil = 30, gridsize = 701^3^):	
–3133.75	2072.99
DelPhi (scale = 6.25, perfil = 30, gridsize = 1459^3^):	
–3126.53	48237.75
DelPhi (scale = 7, perfil = 30, gridsize = 1633^3^):	
–3122.84	78586.87
DelPhi (scale = 9, perfil = 30, gridsize = 2101^3^):	
–3120.85	222556.01
*n*_max_ = 6:	
–3106.19	3.08 (1.04)
*n*_max_ = 10:	
–3109.40	7.32 (2.40)
*n*_max_ = 16:	
–3110.17	30.59 (10.48)
*n*_max_ = 21:	
–3110.17	86.90 (29.59)

a Times indicated in parentheses refer to parallel computing on four parallel workers (see more details in Figure 5).

**Figure 6:**
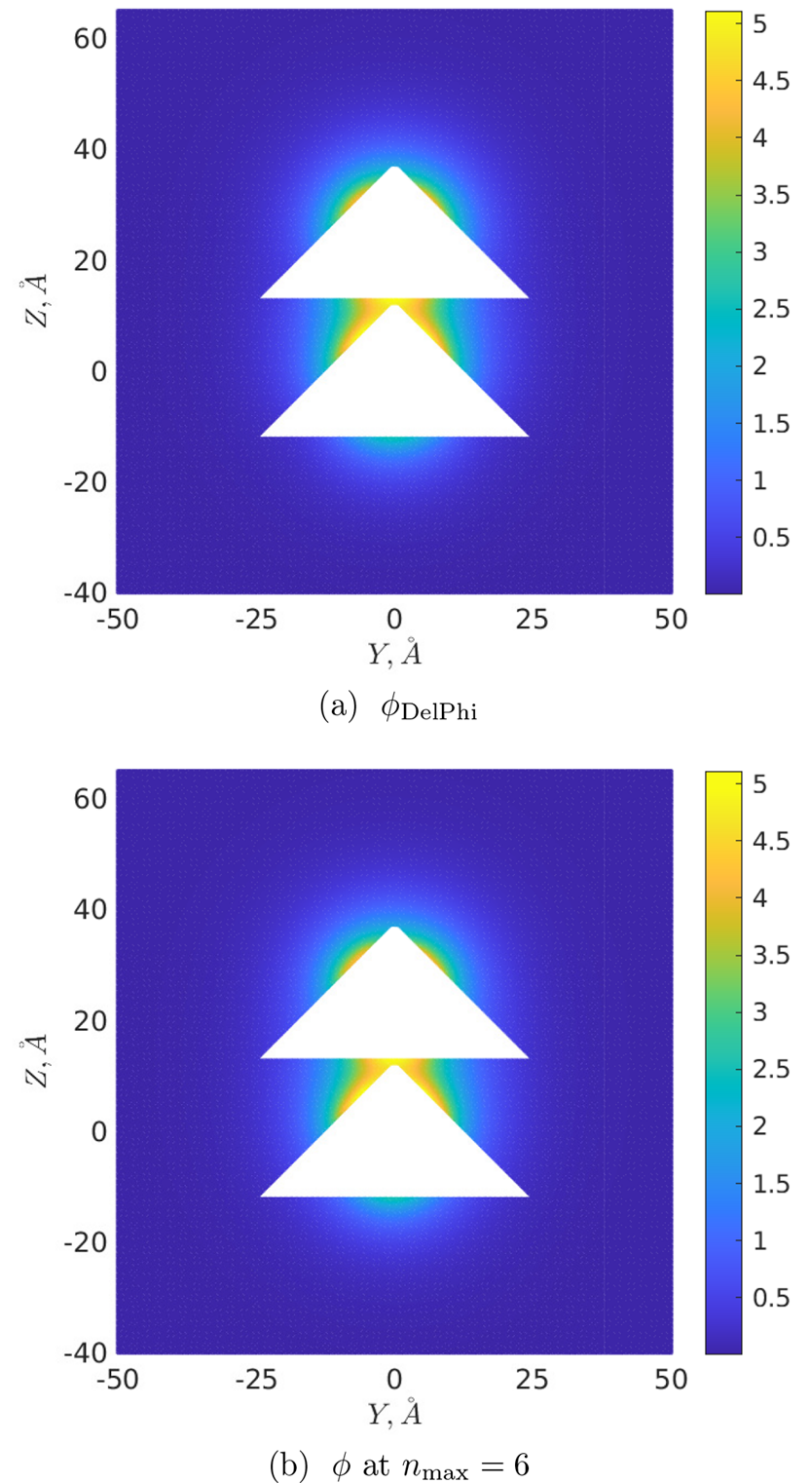
DH potential distribution on the plane *x* = 0, *R* = 25 Å.

**Figure 7:**
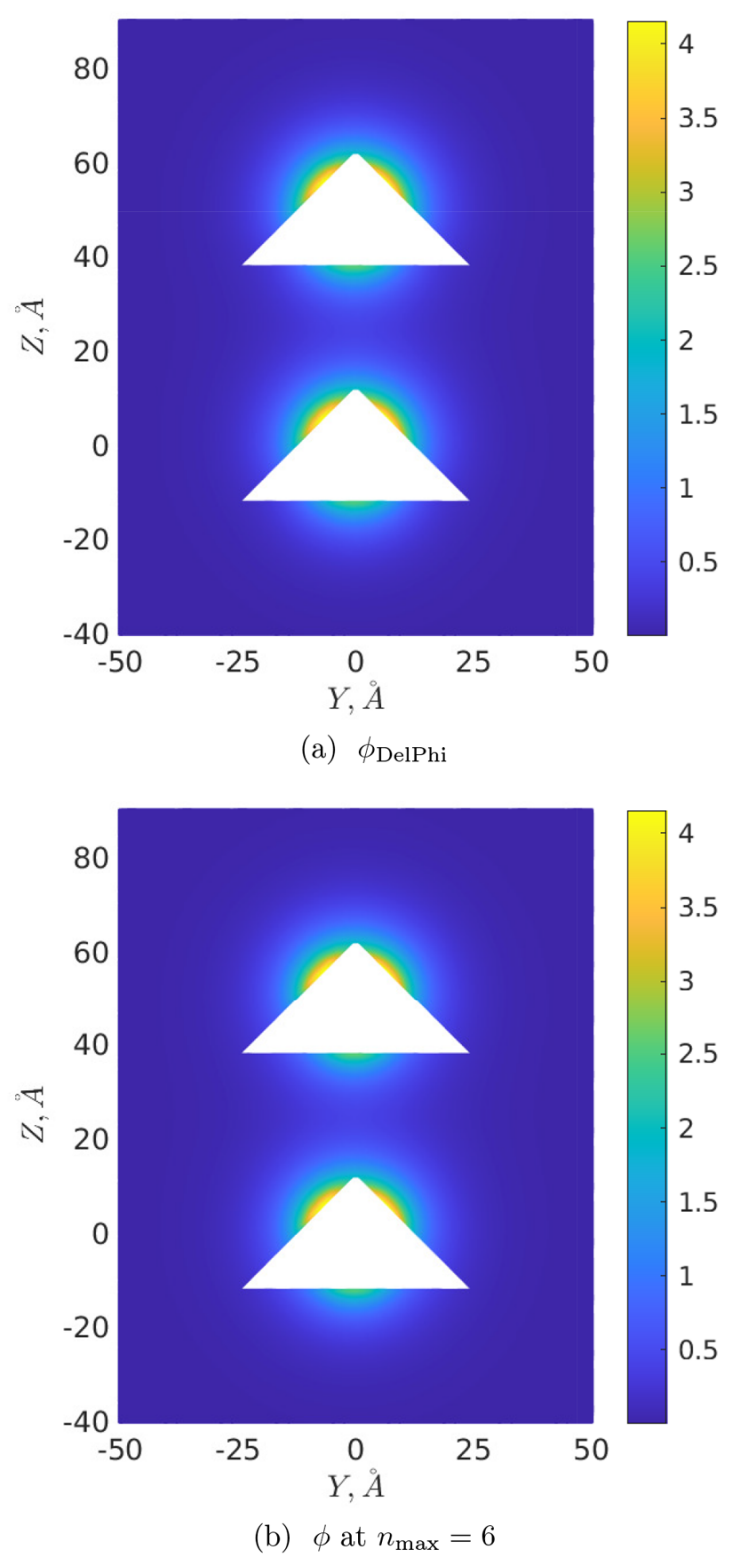
DH potential distribution on the plane *x* = 0, *R* = 50 Å.

Finally, Figures 6 and 7 illustrate the distributions of the corresponding normalized (dimensionless by dividing by *k*_B_*T*/*e*) DH potential  at *R* = 25 Å and *R* = 50 Å, respectively, on the cutting plane *x* = 0. The corresponding “ground-truth” potentials calculated in DelPhi are designated as ϕ_DelPhi_: for their calculation we exploited at maximum the memory capacity of our hardware, leading to the values perfil = 27, scale = 15 for *R* = 25 Å, and perfil = 30, scale = 9 for *R* = 50 Å. To draw Figures 6 and 7, the potentials were determined at points of the mesh Ω_*h*_ at a spacing of 0.25 Å covering the volume [−50 Å, 50 Å]^2^ × [−40 Å, 65 Å] or [−50 Å, 50 Å]^2^ × [−40 Å, 90 Å] around the cones as *R* = 25 Å or *R* = 50 Å, respectively (that is, 30 Å of free space was just added in each coordinate direction around the cones). Furthermore, in order to mitigate any grid effects caused by repositioning the molecular surfaces onto the grid points, these DH potentials were calculated at distances ≥2 Å away from the conical surfaces. One then sees from Figures 6 and 7 that potentials ϕ_DelPhi_ (Figures 6a and 7a) are visually indistinguishable from those calculated by this approach (see Figures 6b and 7b) at *n*_max_ = 6. The corresponding absolute errors max_Ω_*h*__|ϕ – ϕ_DelPhi_| over the whole Ω_*h*_ for *n*_max_ = 6 are 0.28 and 0.20 for *R* = 25 Å and *R* = 50 Å, respectively (an increase in *n*_max_ makes it possible to further reduce these errors, e.g., at *n*_max_ = 10 they become equal to 0.18 and 0.14, respectively).

All integrals 48 and 49 were calculated here using the built-in MATLAB functions (integral/quadgk) with the default MATLAB’s accuracy settings (AbsTol = 10^–10^, RelTol = 10^–6^). Let us also note that the step in *n*_max_ simply implies adding new rows and columns to matrices **A**_*i*;*c*_, **A**_*i*;*s*_, **B**_*i*;*c*_, **B**_*i*;*s*_, **C**_*i*;*c*_, **C**_*i*;*s*_, **D**_*i*;*c*_, **D**_*i*;*s*_, **G**_*i*;*c*_, **G**_*i*;*s*_, **H**_*i*;*c*_, **H**_*i*;*s*_, **I**_*i*;*c*_, **I**_*i*;*s*_, **J**_*i*;*c*_, **J**_*i*;*s*_ and new rows to vectors **M⃗**_*i*;*c*_, **M⃗**_*i*;*s*_, **N⃗**_*i*;*c*_, **N⃗**_*i*;*s*_ (the previously calculated elements remain in their places).

#### 5.1.2 Interacting Cylinders

Let us now briefly consider the application of the developed theory to a system consisting of two cylinders. Two thick cylinders (disks) shown in Figure 2 can be described as
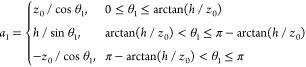
(the similar identity for *a*_2_ can be easily derived by inverting here θ_1_ to π – θ_1_), where 2*z*_0_ is the cylinder height, *z*_0_ = 10 Å, *h* is the cylinder radius, *h* = 50 Å, and two point charges *q*_1_ = *q*_2_ = 10*e* are situated in their centers **x**_1_, **x**_2_.

**Figure 8:**
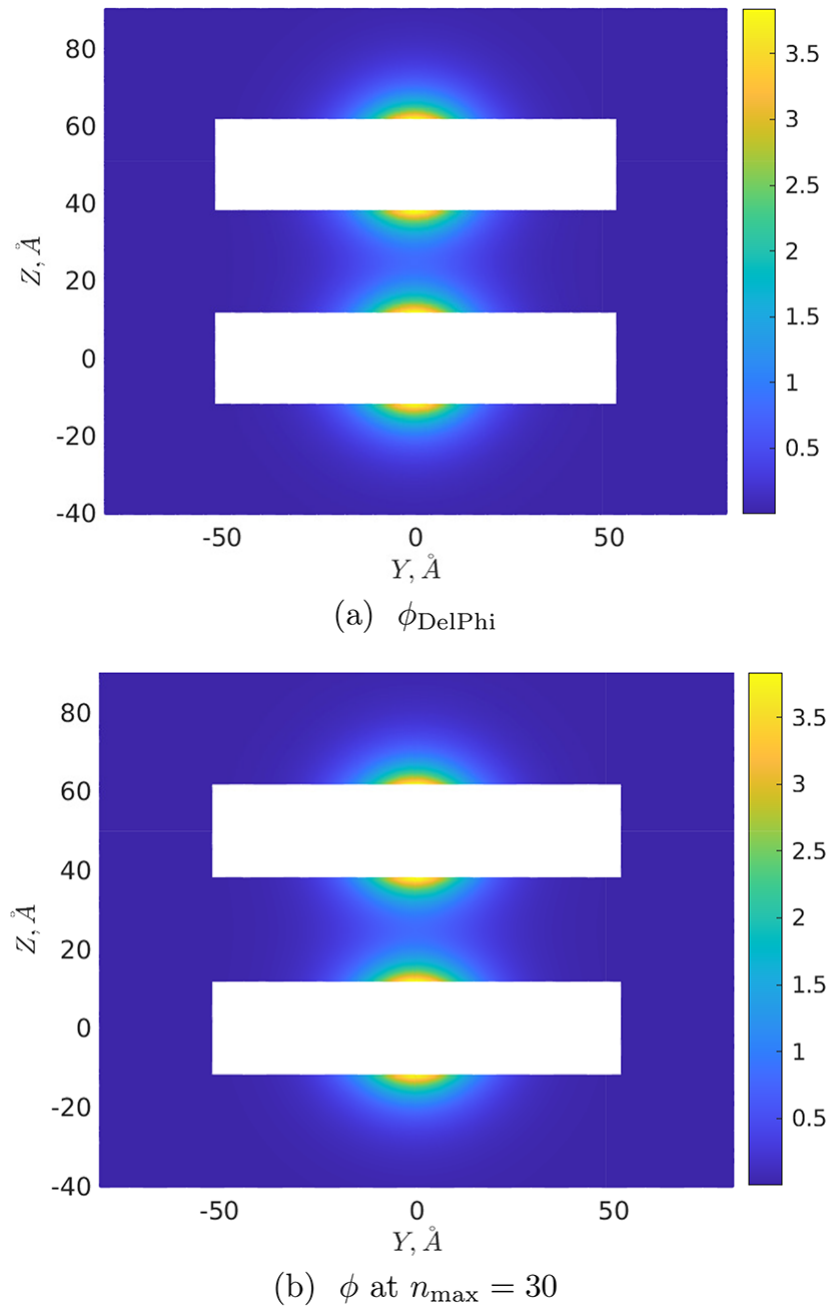
DH potential distribution on the plane *x* = 0, *R* = 50 Å.

As in the previously considered case of a system of two cones (see Figure 4), for sufficiently large *n*_max_, energy  stabilizes and practically stops changing. For instance, in the case shown in Figure 2a (*R* = 50 Å) it happens at *n*_max_ > 25; the corresponding value *k*_B_*T*, whereas DelPhi calculation (with extremely fine parameters scale = 15, perfil = 60, gridsize = 2501^3^) gives *k*_B_*T* in this case. Lastly, Figure 8 illustrates the distributions of the DH potential ϕ calculated by DelPhi with the previously indicated settings (Figure 8a) and using the proposed theory (Figure 8b, *n*_max_ = 30) on the cutting plane *x* = 0. As in the previous case of two cones, the potentials were determined at points of the mesh Ω_*h*_ (which is constructed in the same way as in the case of two cones; see section 5.1.1 for details); the corresponding absolute error max_Ω_*h*__|ϕ – ϕ_DelPhi_| = 0.06. Let us finally note that the computation times of the proposed methodology and DelPhi in this example scale in a similar way to those of the earlier cones example (e.g., the calculation time of the “ground-truth” DelPhi potential shown in Figure 8a took more than 2 days).

#### 5.1.3 Toward More Realistic Systems: Protein Charge Distribution Inside a Geometric Shape

**Figure 9:**
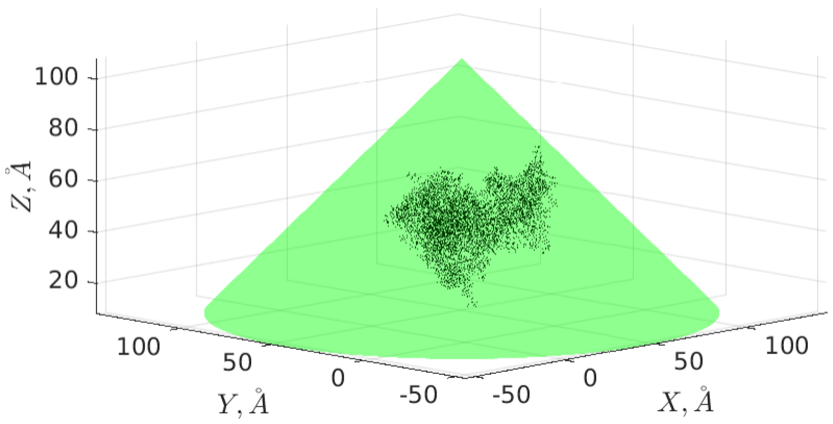
Charge distribution of the *GSK*3β protein placed inside a conical surface.

Let us now consider the charge distribution originating from a realistic protein structure, in this case the glycogen synthase kinase 3 beta (*GSK*3β, pdb code 1J1C) placed inside a conic surface with opening angle π/2 and height 100 Å, centered at the point (45 Å, 45 Å, 58 Å) (see 23 and 15). The corresponding charge distribution consists of 5851 (charged) atoms. This configuration is shown in Figure 9. The total electrostatic energy  of this system calculated in DelPhi (with perfil = 70, scale = 4.5, gridsize = 1285) is −69419.83 *k*_B_*T*, whereas the values provided by the proposed theory are reported in Table 2; it can be seen that already at sufficiently small *n*_max_ equal to 6 and 10, the proposed approach provides values very close to that of Delphi (relative error about 0.01%).

**Table 2:** Energy Calculations for the *GSK*3β Charge Distribution Placed Inside the Cone

*n*_max_	energy, *k*_B_*T*
6	–69412.25
10	–69414.63
20	–69421.02

#### 5.1.4 Showing the Advantage of a Grid-Free Approach: The Potential of Mean Force Estimate

**Figure 10:**
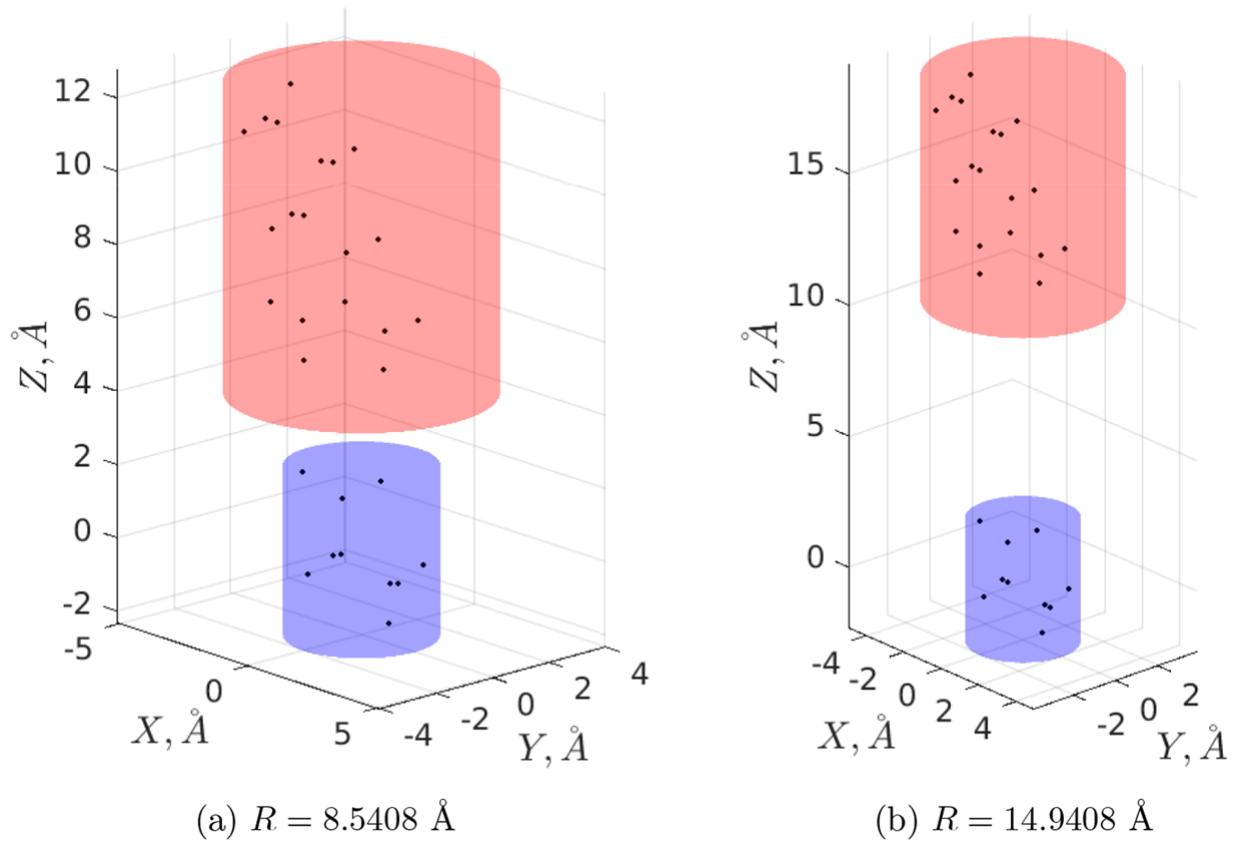
Arginine–glutamate pair (CHARMM22 force field) for extreme values of *R*. Charges are represented as points.

**Figure 11:**
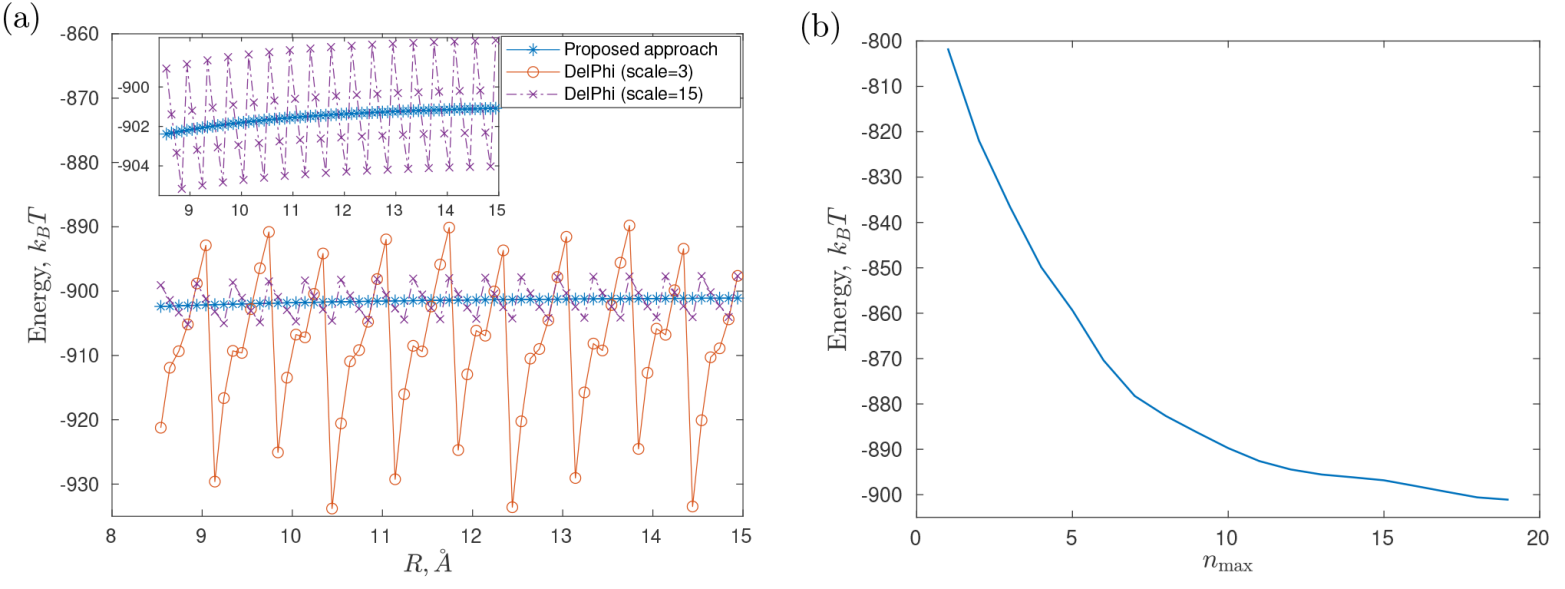
Total electrostatic energy calculations for the arginine–glutamate pair (CHARMM22 force field). (a) The total electrostatic energy profiles for cylinder-embedded arginine and glutamate charge distribution interacting: the proposed approach results (at *n*_max_ = 19) vs those of DelPhi; the embedded inset shows a close-up view. (b) The total electrostatic energy (*R* = 14.9408 Å) at different *n*_max_’s; a further increase in *n*_max_ changes the energy negligibly (by less than 1 *k*_B_*T*).

The experience on grid-based PB solvers taught us to perform energy calculations while preserving the relative position of the system fixed with respect to the grid. As shown in the very simple case of two approaching charged amino acids, see for instance Figure 11 in ref (93), the calculation of the potential of mean force is particularly delicate in this respect since by construction it does not meet the requirement of fixed geometry. Here, we show, as a proof of concept, arginine and glutamate charge distributions placed into two cylindrical dielectric particles (see section 5.1.2 for the corresponding surface parametrizations). We assume that **x**_1_ and **x**_2_ coincide with the corresponding geometric cylinders’ centers and **x**_1_ = (0, 0, 0) (glutamate charge distribution) is fixed while **x**_2_ = (0, 0, *R*) (arginine charge distribution) changes from *R* = 8.54 Å to *R* = 14.94 Å with a step of 0.1 Å (see Figure 10). Then, Figure 11a shows the results of the calculation of the total electrostatic energy  with the proposed methodology and using DelPhi with the parameters perfil = 80, scale = 3, maxc = 10^–5^, which correspond to those used in ref (93); in addition, the results for scale = 15 (which is, in general, an incredibly large value as compared to scales usually used in DelPhi for calculations in systems of biological interest^6,9,93^) and maxc = 10^–7^ are also shown. One can observe that the spurious oscillations pollute the energy profile calculated by DelPhi—these are caused by the numerical (grid) artifacts that are due to the discretization of the equation. In a more realistic case, where the molecular surface would be used rather than a basic geometric model surface, the smallest atomic radius for arginine in the CHARMM parameter set,^93^ which is about 0.22 Å for some polar hydrogens, would even more largely contribute to this phenomenon and require extremely, and practically unfeasible, fine grids.^93^ At the same time, Figure 11a shows that the energy profile calculated by the approach proposed in the current paper is free from such shortcomings. Finally, Figure 11b illustrates the convergence of  as *n*_max_ increases.

### 5.2 Regularizing the Numerical Solution Process of System 14 to Enhance the Stability of Potential Calculations

**Figure 12:**
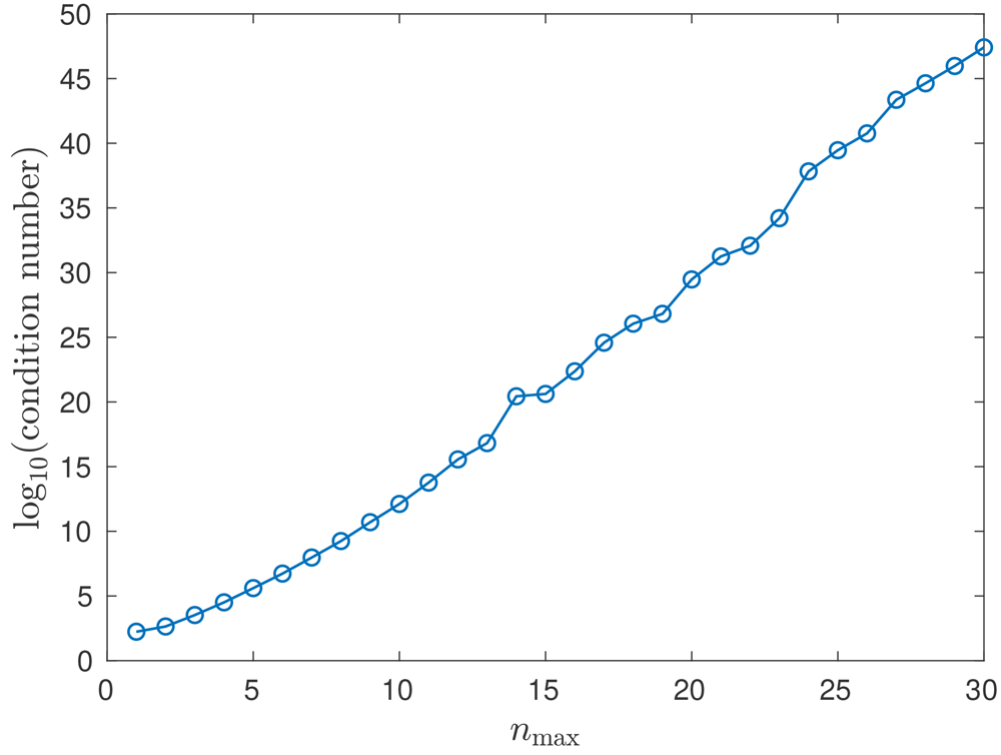
Decimal logarithm of the 2-norm condition number: two interacting cones case (see section 5.1.1), *R* = 50 Å.

The authors of ref (21), which builds the rigorous theory of electrostatic interactions of two spheroids in the azimuthally symmetric case at κ = 0, observed in their calculations that the corresponding linear systems governing the potential coefficients (in our case, system 14) may be ill-conditioned for large *n*_max_ possibly leading to numerical instabilities/artifacts and thence to a loosening of numerical calculations with a further increase in errors in the potential as *n*_max_ increases. For instance, Figure 12 illustrates how the 2-norm condition number (CN) (i.e., the ratio of the largest singular value to the smallest one) of the linear system governing the potential coefficients grows with increasing *n*_max_ in the example with two cones considered above. Let us note that, although CN is a rather rough characteristic, it can still serve as a general indicator of how sensitive a linear system is to numerical errors (inaccuracies that arise when calculating the coefficients of the system, round-off errors in the numerical solution of the system itself, etc.) and how these errors can affect its solution.^94^ Large values of CN indicate that the numerical solution results may be inaccurate/unreliable (e.g., when CN ≳ 10^16^ and double-precision floating-point arithmetic is used). Unfortunately, no specific solution for ill-conditioning was provided in ref (21).

**Figure 13:**
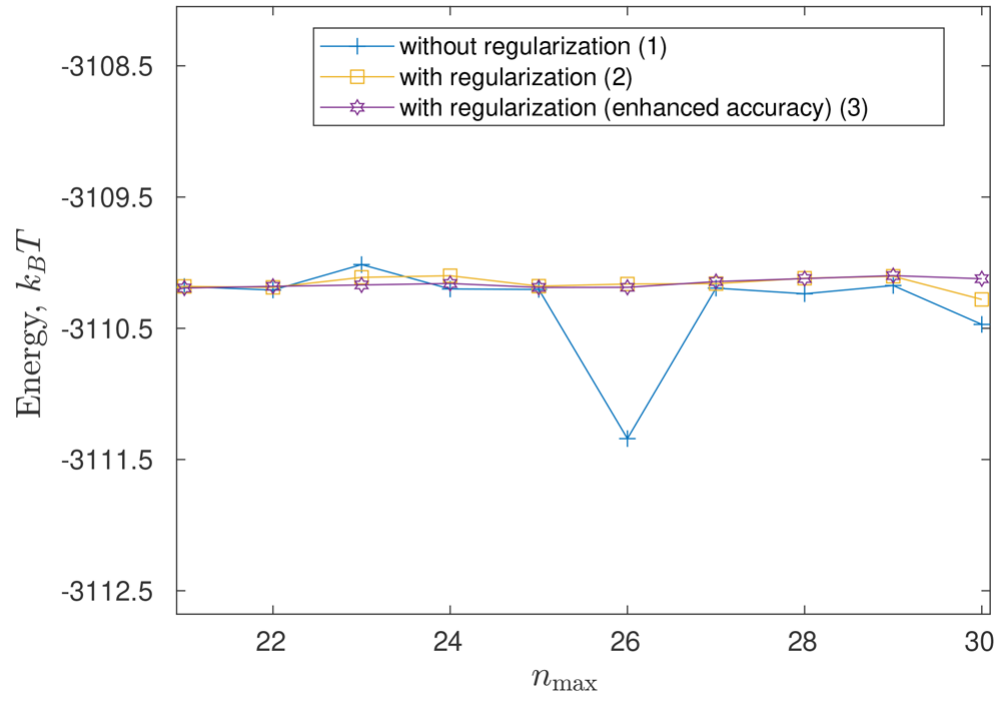
Total electrostatic energy  at higher *n*_max_: two interacting cones (section 5.1.1), *R* = 50 Å. The plot shows the results at different accuracies of calculation of the integrals 48 and 49 by the built-in MATLAB functions integral/quadgk: functions’ default accuracy (AbsTol = 10^–10^, RelTol = 10^–6^) without (line 1) and with (line 2) regularization and one order of enhanced accuracy (AbsTol = 10^–11^, RelTol = 10^–7^) with regularization (line 3) (for the latter accuracy the results without regularization are not drawn, as they are very close to line 1).

Thus, to enhance the stability and robustness of calculations we adopt the following simple approach (which conceptually follows the Tikhonov regularization theory^95^): namely, instead of solving the original (possibly ill-conditioned) system 14 represented here in the matrix form *Ax⃗* = *b⃗* we solve the perturbed system (*A*′*A* + *αE*)*x⃗* = *A*′*b⃗*, where *A*′ denotes the conjugate transpose of *A*, α > 0 is a regularization parameter (so that *x⃗* depends on α now: *x⃗* = *x⃗*_α_), and *E* is some (well-conditioned) symmetric positive-definite regularizer (e.g., the simplest choice for *E*, also tested/used in our numerical experiments, is just the identity matrix). Since the perturbed matrix *A*′*A* + *αE* is symmetric, positive-definite, and well-conditioned (thanks to the regularizing addend *αE*), the corresponding perturbed system can now be effectively handled using, e.g., Cholesky or LDL decompositions.^94^ For the choice of α we followed the idea of the so-called noise level-free quasi-optimality criterion^95−97^ employing the geometric sequence α = α_*i*_ = α_0_*q*^*i*^ (where 0 < *q* < 1, *i* = 1, ..., *M*) and then selecting α_*i*_ which gives the smallest discrepancy  in the 2-norm (or, alternatively, one may also rely on the values of the classical penalized least-squares functional  +  instead; however, in our numerical experiments of section 5 this led to almost the same results). Figure 13 demonstrates the results of such a regularization (we have used α_0_ = 0.8^5^, *q* = 0.8, and *M* = 100 in our numerical experiments) in the problem of two interacting cones (see section 5.1.1) at *R* = 50 Å—it can be seen that the regularized solution behaves in a more stable way (since it is better conditioned). One can also observe from Figure 13 that increasing the accuracy of calculation of the integrals 48 and 49 forming system 14 (as compared to the default accuracy of built-in MATLAB integration functions) as expected improves the numerical solution. We also note that the described regularization procedure could be obviously applied regardless of *n*_max_; however, as our numerical experiments suggest, regularization begins to play a role only for sufficiently large *n*_max_ (e.g., in our numerical experiments, for *n*_max_ > 20; see Figure 13) while for smaller *n*_max_ the regularized and nonregularized solutions practically coincide. At the same time, the computational cost for such a simple regularization is negligibly small compared to the overall time of calculating the integrals 48 and 49 (especially thanks to the small size of the linear system governing the potential coefficients—see the comments on this at the beginning of section 5).

Let us finally note that despite that the numerical solution converges rather rapidly with increasing *n*_max_ (as we can observe from the numerical experiments of section 5.1) and such a simple regularization methodology, considered in the current subsection, usually significantly enhances the stability/reliability of calculations and alleviates the process of numerical solution overall, the (ill-)conditioning of the linear systems governing the potential coefficients may still be a bottleneck issue in the practical computational/numerical applications of the proposed approach and thus needs to be properly addressed in the future studies (beyond the current proof-of-concept analytical work). In this respect, we foresee at least the following two possibilities: (1) investigating better choices for the regularizer *E* and regularizing parameters {α_*i*_} in the current regularization scheme as well as adopting other techniques and rules (see refs (95−98)) for estimating and regularizing the potential solutions; (2) developing *ad hoc* (i.e., specialized for system 14) preconditioners with subsequent usage of iterative methods for solving 14. However, we do not have the possibility to pursue these directions further here.

## 6 Discussion and Conclusions

This paper considers the interaction of two arbitrary-shape polarizable dielectric particles immersed into solvent assuming that the linearized Poisson–Boltzmann equation holds.

In order to rigorously treat the mutual polarization of arbitrary-shape particles at arbitrary distances *R*, in section 3 we present a novel spherical re-expansion for the LPBE solution. Advancing what can be found in the existing literature (refs (2, 12, 19−21, 40, 41, 49−53, 74−81, 99, 100)) neither assumptions on the symmetry of potentials or charge distributions nor on the ratio of *r*_*i*_/*R* are made. Although the obtained general re-expansion coefficients 29 contain infinite series (in contrast to the *r*_*i*_ < *R* case, where these expressions boil down to more compact finite sums 31 and 32), in section 3.2 we propose and discuss an efficient approximation procedure and validate it numerically (Appendix B.3 and section 5).

On this basis, in section 4 we then derive relations governing the potential coefficients (section 4.1). In turn, they then allow us to construct small-parameter (∝ (e^–*κR*^/*R*)^*k*^) asymptotic expansions for the potential coefficients and for the total electrostatic energy in ascending order of Debye screening (section 4.2). These generalize the results established in recent ref (2) for the case of two spherical particles.

Finally, in section 5, we perform the numerical benchmarking of this analytical derivation validating it against the well-known grid-based DelPhi numerical solver^6,9^ on several model examples. Computational examples have been provided with basic shapes, such as cones and cylinders, which can approximate more complex structures at the nanoscale (the general theory built in the article is suitable for arbitrary-shape particles having an analytical representation *a*_*i*_(θ_*i*_, φ), see section 2). Advantages of this approach with respect to conventional grid-based techniques reside in the fact that (i) it is inherently consistent with null boundary conditions for the potential at infinite distance from the solute(s), (ii) its performance is practically independent of the distance *R* between the particles, (iii) being grid-free it is not subjected to numerical artifacts associated with the LPBE discretization or to the presence of the so-called self-energy,^2,6,82^ and (iv) finally, if needed, specific contributions, such as the components arising specifically from the polarization charge at the particle boundaries or ionic contributions can be singled out and studied analytically.^2^ Numerical tests show that the calculation time using the theory proposed in this article can be several orders of magnitude smaller than the corresponding ones in DelPhi. Moreover, a simple parallelization scheme, acting on the assembling process of the elements of system 14, which are governing the potentials, can also be applied—see Figure 5 and corresponding explanations in section 5. Applications of this theory range from a better way of benchmarking numerical grid based approaches for the LPBE, as well as for a better approximation of their boundary conditions,^101^ to allowing a careful study of how geometry impacts on interaction energy, to the treatment of mesoscale systems, approximated as simpler spheroidal or ellipsoidal particles, and their mixtures,^102^ in the fields of both biomolecular modeling, supramolecular assemblies, and colloids. Due to the absence of grid artifacts, this approach appears particularly useful for applications such as the calculation of the potential of mean force, where the same relative position between each of the two particles and the grid can hardly be preserved while the relative distance is changed. Current work is ongoing to instantiate the present formalism in the case of conventional atomistic description of biomolecular systems, such as implementing the various definitions of the protein molecular surface.^93^

## Figures and Tables

**Figure 14:**
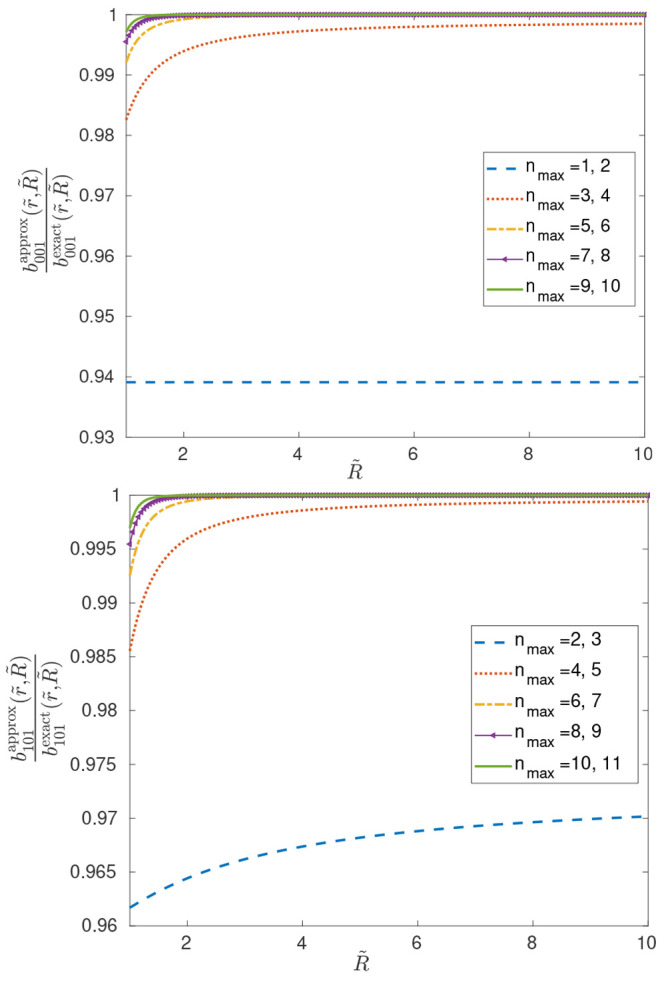
Ratio of the approximated *b*_001_(*r̃*, *R̃*) and *b*_101_(*r̃*, *R̃*) to their exact values given by 38 and 39; *R̃* ≥ *r̃* = 1.

## Data Availability

Coefficients *h*_*kmls*,*n*_ calculated with high precision (and some other related data and code) are openly available in the Zenodo repository at https://doi.org/10.5281/zenodo.6965081.

## A. Derivation the Proposed Novel Re-expansions and the Corresponding Re-Expansion Coefficients for DH Potentials

### A.1 Derivation of Re-expansion 9

Taking into account that

24

where *R̃* ≔ *κR*, , *j* = 3 – *i*, we will use the following Macdonald–Gegenbauer addition theorem (ref (84), Chapter XI):

where denoted , *S̃*_*i*_ ≔ max {*R̃*, *r̃*_*i*_}, *s̃*_*i*_ ≔ min {*R̃*, *r̃*_*i*_}; *I*_ν+*s*_(·) denotes the modified Bessel function of the first kind (Infeld functions), Γ(·) is the Euler Gamma function, *C*_*s*_^ν^ (·) are the Gegenbauer ultraspherical polynomials, and with the corresponding infinite series on the right being absolutely convergent (see ref (84), Chapter XI for details). By letting ν = *l* + 1/2 (with *l* being a non-negative integer) and considering that  =  = , we then arrive at the following identity:

25

(note that (−1)!! = 1 is always assumed^92^).

Further, in order to decompose the product *r̃*_*j*_^*l*^*P*_*l*_^*m*^(μ_*j*_) we also rely on the following decomposition theorem for the associated Legendre polynomials (see ref (85), Chapter IV, eq 24):

26

Let us comment that the original identity is established in ref (85) for the angle π–θ_*i*_ in the argument of *P*_*k*_^*m*^ on the right-hand side, but by using *P*_*k*_^*m*^(−*x*) = (−1)^*k*+*m*^*P*_*k*_^*m*^(*x*) one can easily obtain 26.

Finally, we take advantage of the following representation for the product of a Gegenbauer polynomial with an associated Legendre polynomial:

27

where the numerical coefficients *h*_*kmls*,*n*_ exist and are uniquely determined for arbitrary given polynomials *P*_*k*_^*m*^(*x*), *C*_*s*_^*l*+1/2^(*x*). The representation given in 27, although quite simple, seems to be unnoticed earlier in the literature, and therefore its proof is given in Appendix A.2 alongside with explicit closed-form relations for calculating *h*_*kmls*,*n*_.

Now using 25, 26, and 27, let us now transform the solution Φ_out,*j*_(*r̃*_*j*_, θ_*j*_, φ) given by 7b:

then denoting

28

29

(let us note that *h*_*kmls*,*n*_ = 0 if *s* + *k* – *n* is odd—see Appendix H) and performing transformations of the sums, we finally arrive at representation 9. The double sum in 29 may also be left unbounded if it is understood that all *h*_*kmls*,*n*_ with *n* > *s* + *k* as well as when *s* + *k* – *n* being odd vanish:

30

Note that, by definition, inequalities *n* ≥ *m* and *l* ≥ *m* always hold for the indices of *b*_*nml*_(*r̃*, *R̃*).

Let us also note that re-expansion coefficients 29 generalize the corresponding “azimuthally symmetric” coefficients of refs (19 and 20) (see ref (19), eq 11) in the sense that they recover the latter at *m* = 0 (i.e., in the case of azimuthal symmetry) and *r*_*i*_ < *R*; at the same time, the re-expansion of the azimuthally symmetric potential Φ_out,*j*_(*r̃*_*j*_, μ_*j*_) previously derived in refs (19 and 20) is the particular case of the representation 9 (upon the assumption of the azimuthal symmetry and *r*_*i*_ < *R* conditions). Re-expansion coefficients 29 also generalize (recover at *m* = 0) the “azimuthally symmetric” coefficients of refs (99 and 100). Besides, in the particular case of *r*_*i*_ < *R*, coefficients 29 boil down to equalities previously derived in ref (2), eqs 37 and 40:

31

where Θ_*nml*_(*R̃*) is a polynomial in *R̃*^–1^ of degree *n* + *l* – *m* having as explicit representation

32

The advantage of relations 31 and 32 is that they do not contain any infinite series of modified Bessel functions in their *R*-dependent parts; these relations were derived in ref (2) in a completely different way as compared to the current paper (in ref (2) we have employed Gegenbauer–Sonine identities and the generalized Neumann transforms; however, such a method of proof essentially relied on the condition of *r*_*i*_ < *R*, in contrast to what is done in the current paper). In Appendix B.1 we provide an analytical derivation of exact explicit (closed-form) expressions for some typical values of re-expansion coefficients and compare them with 31, which is also used in testing the proposed numerical methodology for calculating *b*_*nml*_(*r̃*, *R̃*) (see section 3.2).

Finally, let us also note that, in general, the definition 29, in contrast to 31 with 32, does not represent the expansion of *b*_*nml*_(*r̃*, *R̃*) in powers of *R* (due to the analytical representation 68a for *K*_*n*+1/2_(*R̃*)). Nevertheless, using 70 in 28 immediately yields

33

as *r̃* → 0 and *R̃* → 0, *r̃* < *R̃*, *n* ≥ 0; thus, for small values of *r̃* and *R̃* (e.g., in the weak screening regime as κ → 0) one has the relation  ≈ . Employing this approximation for Ω_*l*+*s*+1/2_(*r̃*, *R̃*), the proposed methodology (see section 3.2) allows us to list in 29 all the terms up to (and including) *R*^–(*n*_max_+*m*+1)^.

### A.2 Explicit Construction of the Coefficients *h*_*kmls*,*n*_

We start with the known unique representation of an arbitrary monomial power *x*^*j*^ through Legendre polynomials *P*_*n*_(*x*) (see ref (85)):  ≔ . Differentiating this representation *m* times (*m* ≤ *j*) in *x* and then multiplying the result by (1 – *x*^2^)^*m*/2^, we get to (1 – *x*^2^)^*m*/2^*x*^*j* – *m*^ =  where

Next, since *C*_*s*_^*l*+1/2^(*x*) is a polynomial of degree *s* and *P*_*k*_^*m*^(*x*) = (1 – *x*^2^)^*m*/2^*P̂*_*k*_^*m*^(*x*), where  =  is a polynomial of degree *k* – *m*, then their product  =  with some numerical coefficients  which constitute the polynomial *C*_*s*_^*l*+1/2^(*x*)*P̂*_*k*_^*m*^(*x*) of degree *s* + *k* – *m*. Indeed, the corresponding explicit expressions for the coefficients of the polynomial *P̂*_*k*_^*m*^(*x*) are known (see ref (92), eq 8.812):  = , where  ≔ ··· as *k* ≥ 1, and . Also, using ref (92), eq 8.936(2), we have

34

Using these relations we then end up with the equality

Then

where numerical coefficients *h*_*kmls*,*n*_ are defined as

35

Thus, the explicit closed-form construction provided in 35 completes the proof of representation 27.

Also, additional identities for calculating the coefficients *h*_*kmls*,*n*_ are provided in Appendix H.

## B. Analytical and Numerical Validation of the Re-expansion Coefficients

In this section, we calculate analytically the sums in 29 (for some typical indices, namely, we consider *l* = *m* and *l* = *m* + 1 that are responsible for treating monopoles and dipoles in spherical particles; see ref (2)) and demonstrate their coincidence with 31 and 32 in the case when *r*_*i*_ < *R*. Then, using the exact values of 29, in Appendix B.3 we validate numerically the fast convergence of the approximation methodology of section 3.2.

### B.1 Analytical Summation of Some Re-expansion Coefficients 29 to the Exact Values

It is noteworthy that coefficients *b*_*nmm*_(*r̃*, *R̃*), 0 ≤ *m* ≤ *n*, can be immediately computed using the original definition 29. Indeed, putting *k* = *l* = *m* in 27 and taking into account that  =  (see 34) and *P*_*m*_^*m*^(*x*) = (−1)^*m*^ (1 – *x*^2^)^*m*/2^ (2*m* – 1)!! (see ref (92), eq 8.812), one has *P*_*s* + *m*_^*m*^(*x*) = *P*_*m*_^*m*^(*x*) *C*_*s*_^*m*+1/2^(*x*) = ∑_*n* = *m*_^*s* + *m*^*h*_*mmms,n*_*P*_*n*_^*m*^(*x*); thus, *h*_*mmms,n*_ = δ_*n*_^*s* + *m*^, where δ is a Kronecker delta. Employing this in 29, we immediately arrive at the following:

36

where *s̃* = min(*r̃*, *R̃*), *S̃* = max(*r̃*, *R̃*).

We also take advantage of the following exact equality valid for arbitrary *n* ≥ *m* ≥ 0:

37

The proof of 37, which is rather technical, is left in the separate Appendix B.2. For instance, using 68 one can immediately obtain the following particular cases of 37 as *r̃* < *R̃*:

38

39

It is easy to verify that relations 38, 39, and 36 (at *r̃* < *R̃*) coincide with 31 and 32 previously obtained in ref (2) using different methodology.

### B.2 Proof of Equality 37

Let us prove the equality 37. From 30 we immediately obtain

40

so now we have to determine *h*_*m*,*m*,*m*+1,*s*,*n*_ and *h*_*m*+1,*m*,*m*+1,*s*,*n*_.

(1) Determination of *h*_*m*,*m*,*m*+1,*s*,*n*_: by virtue of 27 we have the relation *P*_*m*_^*m*^(*x*)*C*_*s*_^*m*+1 + 1/2^(*x*) = ∑_*n* = *m*_^*m* + *s*^*h*_*m*, *m*, *m*+1, *s,n*_*P*_*n*_^*m*^(*x*), or using  =  (see ref (92), eq 8.936(2)) and *P*_*m*_^*m*^(*x*) = (−1)^*m*^ (1 – *x*^2^)^*m*/2^ (2*m* – 1)!! (see ref (92), eq 8.812)

On the other hand it is easy to derive from the equality  =  (see ref (92), eq 8.915) that

41

We thus conclude from these identities that *h*_*m*,*m*,*m*+1,*s*,*n*_ = (2*n* + 1)/(2*m* + 1) if *s* + *n* + *m* is even (*m* ≤ *n* ≤ *m* + *s*) and *h*_*m*,*m*,*m*+1,*s*,*n*_ = 0 otherwise. Thus,

42

(2) Determination of *h*_*m*+1,*m*,*m*+1,*s*,*n*_: by virtue of 27 we have *P*_*m*+1_^*m*^(*x*)*C*_*s*_^*m*+1 + 1/2^(*x*) = ∑_*n* = *m*_^*m* + *s* + *1*^*h*_*m*+1,*m*,*m*+1,*s,n*_*P*_*n*_^*m*^(*x*), or using *P*_*m*+1_^*m*^(*x*) = (see ref (92), eq 8.731(2)) = (2*m*+1)*xP*_*m*_^*m*^(*x*) = *x* (−1)^*m*^ (1 – *x*^2^)^*m*/2^ (2*m*+1)!!, the above equality  = , and relation 41

Now using the relation (2*l* + 1)*xP*_*l*_^*m*^(*x*) = (*l* – *m* + 1) *P*_*l*+1_^*m*^(*x*) + (*l* + *m*) *P*_*l* – 1_^*m*^(*x*) (see ref (92), eq 8.731(2)) we conclude that

43

Then, applying the above-derived relations 42 and 43 to 40 we get

44

Representing the last sum in 44 as  =  we will now use the following series to handle the corresponding infinite sum :

45

These series follow directly from 25 (when *r̃* ≔ *r̃*_*i*_, *l* = 0 and μ_*i*_ = cos θ_*i*_ = ± 1 there) and are absolutely convergent. Now using 45 in 44 and performing algebraic transformations we arrive at the desired identity 37.

### B.3 Numerical Validation of the Re-expansion Coefficients Convergence

As an example of the methodology of section 3.2 for approximation of the re-expansion coefficients, Figure 14 provides the corresponding illustrations of the convergence of the calculated values of *b*_001_(*r̃*, *R̃*) and *b*_101_(*r̃*, *R̃*) to the exact values given by 38 and 39. Let us also comment on Figure 14 that, owing to the fact that *h*_*kmls*,*n*_ = 0 if *s* + *n* + *k* is odd (see Appendix H), two adjacent iterations with , , result in the same *b*_001_(*r̃*, *R̃*), and two adjacent iterations with , , result in the same *b*_101_(*r̃*, *R̃*). One can observe in Figure 14 the fast convergence of the re-expansion coefficients approximated by the described methodology to the exact ones. One can also observe in this figure that the discrepancy is larger as *R̃* gets smaller; a further study of the finer convergence properties of the re-expansion coefficients in the vicinity of *R̃* = *r̃* remains to be addressed in future work.

## C. Calculation of **n**_*i*_ · ∇_*r̃*_*i*__*f*(*r̃*_*i*_, θ_*i*_, φ)

The normalized (unit) outward normal vector **n**_*i*_ to the surface *r*_*i*_ = *a*_*i*_(θ_*i*_, φ) of the *i*th particle can be represented as **n**_*i*_ = *n*_*i,r*_(θ_*i*_, φ)**r̂**_*i*_ + *n*_*i*,θ_(θ_*i*_, φ)**θ̂**_*i*_ + *n*_*i*, φ_(θ_*i*_, φ) **φ̂**, where the triple (*n*_*i,r*_, *n*_*i*,θ_, *n*_*i*, φ_) is defined by expression

46

and **r̂**_*i*_, **θ̂**_*i*_, **φ̂** are the local unit spherical basis vectors in the surface’s points. Although expression 46 is not given in widespread handbooks/textbooks, it can easily be derived from the well-known determinant

for the normal vector,^82,92^ substituting there the expressions *x* = *a*_*i*_(θ_*i*_, φ)sin θ_*i*_ cos φ, *y* = *a*_*i*_(θ_*i*_, φ)sin θ_*i*_ sin φ, *z* = *a*_*i*_(θ_*i*_, φ) cos θ_*i*_, and using the standard relations^82^**r̂**_*i*_ = **x̂** sin θ_*i*_ cos φ + **ŷ** sin θ_*i*_ sin φ + **ẑ** cos θ_*i*_, **θ̂**_*i*_ = **x̂** cos θ_*i*_ cos φ + **ŷ** cos θ_*i*_ sin φ – **ẑ** sin θ_*i*_, **φ̂** = −**x̂** sin φ + **ŷ** cos φ.

Then, for a function *f* = *f*(*r̃*_*i*_, θ_*i*_, φ), using  =  +  + , one immediately gets the value of **n**_*i*_ · ∇_*r̃*_*i*__*f* in the surface’s points:

47

with the values *n*_*i*,*r*_, *n*_*i*,θ_, *n*_*i*, φ_ defined by 46. Relation 47 is all we need to unfold the boundary condition 4b, which was actually done in 12 and 49. Note the invariance of 47 to the replacement of θ_*i*_ by π – θ_*i*_; this confirms the validity of 47 for both particles of the configuration considered in the paper (Figure 1).

## D. Coefficients of Systems 11 and 12

Coefficients *a*_*n*′*m*′,*nm*_^*i*; cos^, *b*_*n*′*m*′,*nm*_^*i*; cos^, *c*_*n*′*m*′,*nm*_^*i*; cos^, *d*_*n*′*m*′,*nm*_^*i*; cos^, *e*_*n*′*m*′,*nm*_^*i*; cos^, *f*_*n*′*m*′,*nm*_^*i*; cos^ and *m*_*n*′*m*′_^*i*; cos^ in 11 have the following form:

48

If Φ̂_in,*i*_(*r̃*_*i*_, θ_*i*_, φ) is representable through multipoles 21, the corresponding *m*_*n*′*m*′_^*i*; cos^ takes the following value:

Coefficients *g*_*n*′*m*′,*nm*_^i; cos^, *h*_*n*′*m*′,*nm*_^*i*; cos^, *i*_*n*′*m*′,*nm*_^*i*; cos^, *j*_*n*′*m*′,*nm*_^*i*; cos^, *k*_*n*′*m*′,*nm*_^*i*; cos^, *l*_*n*′*m*′,*nm*_^*i*; cos^ and *n*_*n*′*m*′_^*i*; cos^ in 12 have the following form:

49

If Φ̂_in,*i*_(*r̃*_*i*_, θ_*i*_, φ) is representable through multipoles 21, the corresponding *n*_*n*′*m*′_^*i*; cos^ takes the following value:

### D.1 The Particular Case of a Spherical Surface

In the particular case of a spherical surface, that is *a*_*i*_(θ_*i*_, φ) = constant independently of angles θ_*i*_ and φ, one obtains

50

where δ is a Kronecker delta and factor

is caused by spherical orthogonality relations (see 66); the right-hand side integrals were expressed using 21. The corresponding quantities in 54 then acquire the following values:

51

Employing 51 in 55 we further get

52

where

Then, using relations 52, equations 55 boil down to the following:

53

## E. Explicit Solution of Systems of Equations That Determine Potential Coefficients

Apart from solving the (global) linear system 14 (immediately stemming from the governing relations 13), finding unknown vectors **L**_*i*_, **M**_*i*_, **G**_*i*_, **H**_*i*_,  can be implemented in an alternative way, e.g., one can done it *explicitly* proceeding as follows:

1. Using relations 13a–13b one can express **L**_*i*_ and **M**_*i*_ through remaining vectors **G**_*i*_, **H**_*i*_, **G**_*j*_, **H**_*j*_ (where as usual *j* = 3 – *i*):

54

where we have denoted auxiliary matrices

and vectors

2. Then, substituting 54 into 13c–13d one gets the relations

55a

55b

where we have denoted auxiliary matrices

and vectors

3. Now, for instance, using relation 55b and that of with indices *i* and *j* interchanged, one arrives at the following relation expressing **H**_*i*_ through **G**_*i*_ and **G**_*j*_, where , *j* = 3 – *i*:

56

4. Then, substituting 56 into the remaining relation 55a one finally gets

57

where we have denoted

58

Relation 57 and that of with indices *i* and *j* interchanged immediately yield

59

Now identities 59, 56, and 54 provide explicit (merely resolved in terms of the coefficients of system 14) expressions for **G**_*i*_, **H**_*i*_, **L**_*i*_, **M**_*i*_, .

Let us finally note that, alternatively, instead of obtaining 56, 57, and 58, one may also express **G**_*i*_ via **H**_*i*_ and **H**_*j*_ from 55a, and then substitute it into 55b to obtain an equation relating solely **H**_*i*_ and **H**_*j*_ (completely similar to 57); however, we do not pursue this direction further here.

## F. Re-expansions and Re-expansion Coefficients *b*_*nml*_ as κ → 0, *r̃*_*i*_ < *R̃*

In the limit κ → 0, the linearized homogeneous PBE (see 2) turns into the Laplace equation; thus, instead of potential 7b one gets the corresponding potential satisfying the Laplace equation and having the form

60

Indeed, the right-hand side of 60, with numeric coefficients *G*_*nm,i*_^(lapl)^ and *H*_*nm,i*_^(lapl)^ arbitrarily given, represents a general formal solution to the Laplace equation in the *i*th spherical coordinates (*r*_*i*_, θ_*i*_, φ) vanishing at infinity.^84,85^

The re-expansion of such a solution 60 of the Laplace equation to the spherical coordinates of the opposite sphere was addressed and discussed in refs (21, 54, 58, 103−105) (the particular case of azimuthal symmetry when harmonics with *m* > 0 are not present in 60) and refs (106−108) (general case). The key ingredient for this re-expansion is the following identity (see ref (108), eq B.7, and ref (85), Chapter IV), which is an analogue of equality 26 for negative powers of *r*_*j*_:

61

where *r*_*i*_ < *R*, *r*_*j*_ = , μ_*j*_ = (*R* – *r*_*i*_μ_*i*_)/*r*_*j*_, *i* ∈ {1,2}, *j* = 3 – *i*, and with the corresponding infinite series being absolutely and uniformly convergent. Employing 61 in 60 one easily gets the desired re-expansion:

62

Let us emphasize that 62 is obtained independently of the PBE re-expansion theory built in Appendix A and provides the re-expansion of the Laplace solution of general form 60 with arbitrarily given numeric coefficients {*G*_*nm,i*_^(lapl)^} and {*H*_*nm,i*_^(lapl)^}.

As well, multiplying expansion 61 by *P*_*n*_^*m*^, denoting *z* = *r*_*i*_/*R* < 1 there, integrating term by term (it is permitted because 61 is uniformly convergent) and using the orthogonality property 66, we arrive at the following useful integral (ultimately valid for an arbitrary real number *z* ∈ (0, 1)):

63

(Let us point out the nontriviality of integral 63—see ref (109) especially devoted to its calculation, where it was obtained in a different and more complicated way.)

Let us now examine how the PBE re-expansion theory built in Appendix A matches that for the corresponding (simpler, in general) Laplace case as κ → 0 (accordingly, then *r̃*_*i*_ = *κr*_*i*_ → 0 and *R̃* = *κR* → 0 for finite *r*_*i*_ and *R*). Employing the approximation 70 ≈  =  for small *r̃*_*i*_ = *κr*_*i*_ → 0 in 7b, one gets the formal approximation Φ_out,*i*_(*r̃*_*i*_, θ_*i*_, φ) ≈ Φ_out,*i*_^(lapl)^(*r*_*i*_, θ_*i*_, φ) for potential 7b, where Φ_out,*i*_^(lapl)^ is given by 60 with the coefficients related as *G*_*nm,i*_^(lapl)^ = , *H*_*nm,i*_^(lapl)^ = . Thus, since potentials 7b and 60 possess such a relationship, one may anticipate the corresponding re-expansions to match up as well, namely, re-expansion 9 of potential 7b should approach re-expansion 62 of potential 60. Hence, by simple comparison of the re-expansions 9 and 62 (taken with the above coefficients *G*_*nm,i*_^(lapl)^ and *H*_*nm,i*_^(lapl)^) one would expect to have the following asymptotics for small κ → 0:

64

Relation 64 constitutes the key result of this Appendix. Let us now prove rigorously that the right-hand side of 64 indeed follows directly from the original definition of *b*_*nml*_(*r̃*, *R̃*) (see 29 and 30) if equivalence 33 is used there to approximate Ω_*n*+1/2_(*r̃*, *R̃*). Indeed, from 30 one then has

In the particular case *m* = 0 (azimuthal symmetry) relation 64 recovers ref (21), eq 6, which being then employed in the azimuthally symmetric counterpart of system 14 for the interaction of two spheroids yields the relations derived in ref (21), section II C.

## G. The Case of Using Complex-Valued Spherical Harmonics to Represent Potentials

Many authors^40,41,51,78^ prefer to express potentials 7 in terms of complex-valued spherical harmonics *Y*_*nm*_(θ_*i*_, φ) instead of using the real-valued ones; e.g., this makes sense if one wants to take advantage of the simplicity of the rotation of spherical harmonics using the Wigner functions^51,78^ (which, in turn, allows one to re-expand the external potentials in multibody systems^78^). It can be shown that re-expansion 9 can be rewritten for this case in terms of *Y*_*nm*_ as follows:

65

where *b̆*_*nml*_ (*r̃*_*i*_,*R̃*) ≔ , and {*G*_*nm,j*_} are arbitrary expansion coefficients with the usual condition *G*_*n*,–*m,j*_ = (−1)^*m*^*G*_*nm,j*_^★^ (the star ^★^ denotes the complex conjugation) ensuring that Φ_out,*j*_ is real-valued. The proof of 65 is the following:

## H. Alternative Expressions for the Expansion Coefficients

Associated Legendre polynomials possess the orthogonality property^92^

66

where 0 ≤ *m* ≤ *n* and 0 ≤ *m* ≤ *n*_1_. Although the explicit theoretical construction described in Appendix A.2, in principle, completely determines the coefficients {*h*_*kmls,n*_} in 27, using equality

67

which immediately follows from the orthogonality property 66 recast to 27 might be easier for practical calculations, since for given particular values of the indices the last integral is readily computable analytically by any computer algebra system. However, to the best of our knowledge, the integral 67 is unavailable in the literature, so let us find the explicit expression for 67 in a closed form. Due to the oddness of the integrand in 67, *h*_*kmls*,*n*_ = 0 if *s* + *n* + *k* is odd. Let us consider the situation when *s* + *n* + *k* is even. Using ref (110), eq 18, we have

where for the summation |*n* – *k*| ≤ *p* ≤ *n* + *k*, *p* ≥ 2*m*, *p* + *n* + *k* is even, and *C*_*m,m*,2*m*_^*n,k,p*^, *C*_0,0,0_^*n,k,p*^ are Clebsch–Gordan coefficients.^111^ It is worth noting that using the Wigner 3-*j* symbols^111^ (for the calculation of which many computer algebra systems have built-in procedures, e.g., Wolfram Mathematica),  can also be rewritten as

Then utilizing ref (112), eqs 16, 19, we arrive at the following explicit closed-form expression for 67:

where we denoted

and _4_*F*_3_ is the generalized hypergeometric function^92,112^ of unit argument that is available for calculation by the built-in procedures in most computer algebra systems. Moreover, it was shown in ref (112) that the corresponding hypergeometric series is a terminating one if *l*, *m*, *k*, *n* are non-negative integers and Re((*m* + *n*)/2 – *r*) > 0, which is valid in our case.

## I. Modified Bessel Functions *K*_*n*+1/2_(·) and *I*_*n*+1/2_(·)

Functions *K*_*n*+1/2_(·) and *I*_*n*+1/2_(·) of semi-integer order *n* + 1/2 have the following exact analytic representations:^84,92^

68a

68b

For further convenience, we also recall the first few functions: *K*_1/2_(*x*) = , *I*_1/2_(*x*) = , *K*_3/2_(*x*) = , *I*_3/2_(*x*) = . Also, there are the following formulas for derivatives:^84^

69

For small *x* → 0^+^ one has^84^

70

while for large *x* → + ∞ one has

(notation “*f*(*x*) ∼ *g*(*x*) as *x* → *y*” here means that *f*(*x*) behaves asymptotically like *g*(*x*) as *x* → *y*).

## References

[ref1] Sheinerman F. B.; Norel R.; Honig B. Electrostatic aspects of protein protein interactions. Curr. Opin. Struct. Biol. 2000, 10, 153–159. 10.1016/S0959-440X(00)00065-8. 10753808

[ref2] Siryk S. V.; Bendandi A.; Diaspro A.; Rocchia W. Charged dielectric spheres interacting in electrolytic solution: a linearized Poisson-Boltzmann equation model. J. Chem. Phys. 2021, 155, 114114 10.1063/5.0056120. 34551534

[ref3] Decherchi S.; Masetti M.; Vyalov I.; Rocchia W. Implicit solvent methods for free energy estimation. Eur. J. Med. Chem. 2015, 91, 27–42. 10.1016/j.ejmech.2014.08.064. 25193298 PMC4310817

[ref4] Ringe S.; Oberhofer H.; Hille C.; Matera S.; Reuter K. Function-space-based solution scheme for the size-modified Poisson-Boltzmann equation in full-potential DFT. J. Chem. Theory Comput. 2016, 12, 4052–4066. 10.1021/acs.jctc.6b00435. 27323006

[ref5] Tabrizi A. M.; Goossens S.; Rahimi A. M.; Cooper C. D.; Knepley M. G.; Bardhan J. P. Extending the solvation-layer interface condition continum electrostatic model to a linearized Poisson-Boltzmann solvent. J. Chem. Theory Comput. 2017, 13, 2897–2914. 10.1021/acs.jctc.6b00832. 28379697

[ref6] Rocchia W.; Alexov E.; Honig B. Extending the applicability of the nonlinear Poisson-Boltzmann equation: multiple dielectric constants and multivalent ions. J. Phys. Chem. B 2001, 105, 6507–6514. 10.1021/jp010454y.

[ref7] Materese C. K.; Savelyev A.; Papoian G. A. Counterion atmosphere and hydration patterns near a nucleosome core particle. J. Am. Chem. Soc. 2009, 131, 15005–15013. 10.1021/ja905376q. 19778017

[ref8] Izadi S.; Anandakrishnan R.; Onufriev A. V. Implicit solvent model for million-atom atomistic simulations: insights into the organization of 30-nm chromatin fiber. J. Chem. Theory Comput. 2016, 12, 5946–5959. 10.1021/acs.jctc.6b00712. 27748599 PMC5649046

[ref9] Rocchia W.; Sridharan S.; Nicholls A.; Alexov E.; Chiabrera A.; Honig B. Rapid grid-based construction of the molecular surface and the use of induced surface charge to calculate reaction field energies: Applications to the molecular systems and geometric objects. J. Comput. Chem. 2002, 23, 128–137. 10.1002/jcc.1161. 11913378

[ref10] Jurrus E.; Engel D.; Star K.; Monson K.; Brandi J.; Felberg L. E.; Brookes D. H.; Wilson L.; Chen J.; Liles K.; et al. Improvements to the APBS biomolecular solvation software suite. Protein Sci. 2018, 27, 112–128. 10.1002/pro.3280. 28836357 PMC5734301

[ref11] Debye P.; Hückel E. Zur Theorie der Elektrolyte. I. Gefrierpunktserniedrigung und verwandte Erscheinungen. Phys. Z. 1923, 24, 185–206. 10.1007/BFb0111753.

[ref12] Fisher M. E.; Levin Y.; Li X. The interaction of ions in an ionic medium. J. Chem. Phys. 1994, 101, 2273–2282. 10.1063/1.467668.

[ref13] Leroy P.; Maineult A. Exploring the electrical potential inside cylinders beyond the Debye-Hückel approximation: a computer code to solve the Poisson-Boltzmann equation for multivalent electrolytes. Geophys. J. Int. 2018, 214, 58–69. 10.1093/gji/ggy124.

[ref14] Ohshima H. Approximate expression for the potential energy of the double-layer interaction between two parallel ion-penetrable membranes at small separations in an electrolyte solution. J. Colloid Interface Sci. 2010, 350, 249–252. 10.1016/j.jcis.2010.06.044. 20663511

[ref15] Iglesias J. A.; Nakov S. Weak formulations of the nonlinear Poisson-Boltzmann equation in biomolecular electrostatics. Journal of Mathematical Analysis and Applications 2022, 511, 126065 10.1016/j.jmaa.2022.126065.

[ref16] Chen C.; Yu B.; Yousefi R.; Iwahara J.; Pettitt B. Assessment of the components of the electrostatic potential of proteins in solution: comparing experiment and theory. J. Phys. Chem. B 2022, 126, 4543–4554. 10.1021/acs.jpcb.2c01611. 35696448 PMC9832648

[ref17] McQuarrie D. A. Statistical Mechanics; Harper and Row: New York, 1976.

[ref18] Israelachvili J. N. Intermolecular and Surface Forces, 3rd ed.; Elsevier Inc.: Waltham, MA, 2011.

[ref19] Derbenev I. N.; Filippov A. V.; Stace A. J.; Besley E. Electrostatic interactions between charged dielectric particles in an electrolyte solution. J. Chem. Phys. 2016, 145, 084103 10.1063/1.4961091. 27586900

[ref20] Derbenev I. N.; Filippov A. V.; Stace A. J.; Besley E. Electrostatic interactions between charged dielectric particles in an electrolyte solution: constant potential boundary conditions. Soft Matter 2018, 14, 5480–5487. 10.1039/C8SM01068D. 29926874

[ref21] Derbenev I. N.; Filippov A. V.; Stace A. J.; Besley E. Electrostatic interactions between spheroidal dielectric particles. J. Chem. Phys. 2020, 152, 024121 10.1063/1.5129756. 31941309

[ref22] Samaj L.; Trizac E. Effective charge of cylindrical and spherical colloids immersed in an electrolyte: the quasi-planar limit. Journal of Physics A: Mathematical and Theoretical 2015, 48, 265003 10.1088/1751-8113/48/26/265003.

[ref23] Momot A. I.; Zagorodny A. G.; Orel I. S. Interaction force between two finite-size charged particles in weakly ionized plasma. Phys. Rev. E 2017, 95, 013212 10.1103/PhysRevE.95.013212. 28208425

[ref24] Momot A. I. Effective charge of a macroparticle in a non-isothermal plasma within the Poisson-Boltzmann model. Contributions to Plasma Physics 2018, 58, 233–238. 10.1002/ctpp.201700074.

[ref25] Dolinnyi A. I. Effective parameters of charged spherical particles in 1:1 electrolyte solutions. Colloid J. 2020, 82, 661–671. 10.1134/S1061933X20060034.

[ref26] Alexander S.; Chaikin P. M.; Grant P.; Morales G. J.; Pincus P.; Hone D. J. Charge renormalization, osmotic pressure, and bulk modulus of colloidal crystals: Theory. J. Chem. Phys. 1984, 80, 5776–5781. 10.1063/1.446600.

[ref27] Trizac E.; Bocquet L.; Aubouy M.; von Grunberg H. H. Alexander’s prescription for colloidal charge renormalization. Langmuir 2003, 19, 4027–4033. 10.1021/la027056m.

[ref28] Alvarez C.; Tellez G. Screening of charged spheroidal colloidal particles. J. Chem. Phys. 2010, 133, 144908 10.1063/1.3486558. 20950042

[ref29] Gillespie D. A. J.; Hallett J. E.; Elujoba O.; Hamzah A. F. C.; Richardson R. M.; Bartlett P. Counterion condensation on spheres in the salt-free limit. Soft Matter 2014, 10, 566–577. 10.1039/C3SM52563E. 24651922

[ref30] Nikam R.; Xu X.; Kanduc M.; Dzubiella J. Competitive sorption of monovalent and divalent ions by highly charged globular macromolecules. J. Chem. Phys. 2020, 153, 044904 10.1063/5.0018306. 32752704

[ref31] Tang Q.; Rubinstein M. Where in the world are condensed counterions?. Soft Matter 2022, 18, 1154–1173. 10.1039/D1SM01494C. 35024721 PMC8965743

[ref32] Xu X.; Ran Q.; Haag R.; Ballauff M.; Dzubiella J. Charged dendrimers revisited: effective charge and surface potential of dendritic polyglycerol sulfate. Macromolecules 2017, 50, 4759–4769. 10.1021/acs.macromol.7b00742.

[ref33] Yuan H.; Deng W.; Zhu X.; Liu G.; Craig V. S. J. Colloidal systems in concentrated electrolyte solutions exhibit re-entrant long-range electrostatic interactions due to underscreening. Langmuir 2022, 38, 6164–6173. 10.1021/acs.langmuir.2c00519. 35512818 PMC9119301

[ref34] Lindgren E.; Quan C.; Stamm B. Theoretical analysis of screened many-body electrostatic interactions between charged polarizable particles. J. Chem. Phys. 2019, 150, 044901 10.1063/1.5079515. 30709241

[ref35] Nakov S.; Sobakinskaya E.; Renger T.; Kraus J. ARGOS: An adaptive refinement goal-oriented solver for the linearized Poisson-Boltzmann equation. J. Comput. Chem. 2021, 42, 1832–1860. 10.1002/jcc.26716. 34302374

[ref36] Amadu M.; Miadonye A. Applicability of the linearized Poisson-Boltzmann theory to contact angle problems and application to the carbon dioxide-brine-solid systems. Sci. Rep. 2022, 12, 5710 10.1038/s41598-022-09178-w. 35383219 PMC8983767

[ref37] Wilson L.; Krasny R. Comparison of the MSMS and NanoShaper molecular surface triangulation codes in the TABI Poisson-Boltzmann solver. J. Comput. Chem. 2021, 42, 1552–1560. 10.1002/jcc.26692. 34041777

[ref38] Benner P.; Khoromskaia V.; Khoromskij B.; Kweyu C.; Stein M. Regularization of Poisson-Boltzmann type equations with singular source terms using the range-separated tensor format. SIAM J. Sci. Comp. 2021, 43, A415–A445. 10.1137/19M1281435.

[ref39] Search S. D.; Cooper C. D.; van’t Wout E. Towards optimal boundary integral formulations of the Poisson-Boltzmann equation for molecular electrostatics. J. Comput. Chem. 2022, 43, 674–691. 10.1002/jcc.26825. 35201634

[ref40] Yu Y.-K. Electrostatics of charged dielectric spheres with application to biological systems. III. Rigorous ionic screening at the Debye-Hückel level. Phys. Rev. E 2020, 102, 052404 10.1103/PhysRevE.102.052404. 33327080 PMC10510731

[ref41] Obolensky O. I.; Doerr T. P.; Yu Y.-K. Rigorous treatment of pairwise and many-body electrostatic interactions among dielectric spheres at the Debye-Hückel level. Eur. Phys. J. E 2021, 44, 129 10.1140/epje/s10189-021-00131-9. 34661792 PMC8523465

[ref42] Silva G. M.; Liang X.; Kontogeorgis G. M. Investigation of the limits of the linearized Poisson-Boltzmann equation. J. Phys. Chem. B 2022, 126, 4112–4131. 10.1021/acs.jpcb.2c02758. 35623090

[ref43] Wilson L.; Geng W.; Krasny R. TABI-PB 2.0: An improved version of the treecode-accelerated boundary integral Poisson-Boltzmann solver. J. Phys. Chem. B 2022, 126, 7104–7113. 10.1021/acs.jpcb.2c04604. 36101978

[ref44] Urzua S. A.; Sauceda-Olono P. Y.; Garcia C. D.; Cooper C. D. Predicting the orientation of adsorbed proteins steered with electric fields using a simple electrostatic model. J. Phys. Chem. B 2022, 126, 5231–5240. 10.1021/acs.jpcb.2c03118. 35819287

[ref45] Filippov A. V. Effect of the size of macroparticles on their electrostatic interaction in a plasma. Journal of Experimental and Theoretical Physics 2009, 109, 516–529. 10.1134/S1063776109090179.

[ref46] Li X.; Levin Y.; Fisher M. E. Cavity forces and criticality in electrolytes. Europhys. Lett. 1994, 26, 683–688. 10.1209/0295-5075/26/9/008.

[ref47] Phillies G. D. J. Excess chemical potential of dilute solutions of spherical polyelectrolytes. J. Chem. Phys. 1974, 60, 2721–2731. 10.1063/1.1681434.

[ref48] Sushkin N. V.; Phillies G. D. J. Charged dielectric spheres in electrolyte solutions: Induced dipole and counterion exclusion effects. J. Chem. Phys. 1995, 103, 4600–4612. 10.1063/1.470647.

[ref49] Carnie S.; Chan D. Interaction free energy between identical spherical colloidal particles: the linearized Poisson-Boltzmann theory. J. Colloid Interface Sci. 1993, 155, 297–312. 10.1006/jcis.1993.1039.

[ref50] Stankovich J.; Carnie S. L. Interactions between two spherical particles with nonuniform surface potentials: the linearized Poisson-Boltzmann theory. J. Colloid Interface Sci. 1999, 216, 329–347. 10.1006/jcis.1999.6326. 10421741

[ref51] McClurg R. B.; Zukoski C. F. The electrostatic interaction of rigid, globular proteins with arbitrary charge distributions. J. Colloid Interface Sci. 1998, 208, 529–542. 10.1006/jcis.1998.5858. 9845697

[ref52] Ohshima H.; Mishonova E.; Alexov E. Electrostatic interaction between two charged spherical molecules. Biophys. Chem. 1996, 57, 189–203. 10.1016/0301-4622(95)00056-1. 17023339

[ref53] Bozic A. L.; Podgornik R. Symmetry effects in electrostatic interactions between two arbitrarily charged spherical shells in the Debye-Hückel approximation. J. Chem. Phys. 2013, 138, 074902 10.1063/1.4790576. 23445030

[ref54] Bichoutskaia E.; Boatwright A. L.; Khachatourian A.; Stace A. J. Electrostatic analysis of the interactions between charged particles of dielectric materials. J. Chem. Phys. 2010, 133, 024105 10.1063/1.3457157. 20632746

[ref55] Lindgren E. B.; Chan H.-K.; Stace A. J.; Besley E. Progress in the theory of electrostatic interactions between charged particles. Phys. Chem. Chem. Phys. 2016, 18, 5883–5895. 10.1039/C5CP07709E. 26841284

[ref56] Lian H.; Qin J. Polarization energy of two charged dielectric spheres in close contact. Molecular Systems Design and Engineering 2018, 3, 197–203. 10.1039/C7ME00105C.

[ref57] Chan H.-K. A theory for like-charge attraction of polarizable ions. J. Electrost. 2020, 105, 103435 10.1016/j.elstat.2020.103435.

[ref58] Linden F.; Cederquist H.; Zettergren H. Interaction and charge transfer between dielectric spheres: Exact and approximate analytical solutions. J. Chem. Phys. 2016, 145, 194307 10.1063/1.4967701. 27875888

[ref59] Qin J. Charge polarization near dielectric interfaces and the multiple-scattering formalism. Soft Matter 2019, 15, 2125–2134. 10.1039/C8SM02196A. 30762054

[ref60] Lian H.; Qin J. Exact polarization energy for clusters of contacting dielectrics. Soft Matter 2022, 18, 6411–6418. 10.1039/D2SM00245K. 35979741

[ref61] Stace A. J.; Boatwright A. L.; Khachatourian A.; Bichoutskaia E. Why like-charged particles of dielectric materials can be attracted to one another. J. Colloid Interface Sci. 2011, 354, 417–420. 10.1016/j.jcis.2010.11.030. 21131001

[ref62] Xu Z. Electrostatic interaction in the presence of dielectric interfaces and polarization-induced like-charge attraction. Phys. Rev. E 2013, 87, 013307 10.1103/PhysRevE.87.013307. 23410460

[ref63] Larsen A. E.; Grier D. G. Like-charge attractions in metastable colloidal crystallites. Nature 1997, 385, 230–233. 10.1038/385230a0.

[ref64] Levin Y. When do like charges attract?. Physica A: Statistical Mechanics and its Applications 1999, 265, 432–439. 10.1016/S0378-4371(98)00552-4.

[ref65] Todd B. A.; Eppell S. J. Probing the limits of the Derjaguin approximation with scanning force microscopy. Langmuir 2004, 20, 4892–4897. 10.1021/la035235d. 15984247

[ref66] Zhou S. Investigation about validity of the Derjaguin approximation for electrostatic interactions for a sphere-sphere system. Colloid Polym. Sci. 2019, 297, 623–631. 10.1007/s00396-019-04469-7.

[ref67] Khachatourian A.; Chan H. K.; Stace A. J.; Bichoutskaia E. Electrostatic force between a charged sphere and a planar surface: A general solution for dielectric materials. J. Chem. Phys. 2014, 140, 074107 10.1063/1.4862897. 24559338

[ref68] Gomez-Flores A.; Bradford S. A.; Wu L.; Kim H. Interaction energies for hollow and solid cylinders: Role of aspect ratio and particle orientation. Colloids Surf., A 2019, 580, 123781 10.1016/j.colsurfa.2019.123781.

[ref69] Wu L.; Gao B.; Tian Y.; Munoz-Carpena R.; Zigler K. J. DLVO interactions of carbon nanotubes with isotropic planar surfaces. Langmuir 2013, 29, 3976–3988. 10.1021/la3048328. 23442014

[ref70] Stolarczyk J. K.; Sainsbury T.; Fitzmaurice D. Evaluation of interactions between functionalised multi-walled carbon nanotubes and ligand-stabilised gold nanoparticles using surface element integration. Journal of Computer-Aided Materials Design 2007, 14, 151–165. 10.1007/s10820-006-9027-8.

[ref71] Bhattacharjee S.; Elimelech M. Surface element integration: a novel technique for evaluation of DLVO interaction between a particle and a flat plate. J. Colloid Interface Sci. 1997, 193, 273–285. 10.1006/jcis.1997.5076. 9344528

[ref72] Bhattacharjee S.; Chen J. Y.; Elimelech M. DLVO interaction energy between spheroidal particles and a flat surface. Colloids and SurfacesA: Physicochemical and Engineering Aspects 2000, 165, 143–156. 10.1016/S0927-7757(99)00448-3.

[ref73] Folescu D.; Onufriev A. A closed-form, analytical approximation for apparent surface charge and electric field of molecules. ACS Omega 2022, 7, 26123–26136. 10.1021/acsomega.2c01484. 35936397 PMC9352323

[ref74] Glendinning A. B.; Russel W. B. The electrostatic repulsion between charged spheres from exact solutions to the linearized Poisson-Boltzmann equation. J. Colloid Interface Sci. 1983, 93, 95–104. 10.1016/0021-9797(83)90388-0.

[ref75] Clercx H. J. H.; Schram P. P. J. M. An alternative expression for the addition theorems of spherical wave solutions of the Helmholtz equation. Journal of Mathematical Physics 1993, 34, 5292–5301. 10.1063/1.530305.

[ref76] Langbein D. Theory of van der Waals Attraction; Springer: Berlin, Germany, 1974.

[ref77] Ether D. S.; Rosa F. S. S.; Tibaduiza D. M.; Pires L. B.; Decca R. S.; Neto P. A. M. Double-layer force suppression between charged microspheres. Phys. Rev. E 2018, 97, 022611 10.1103/PhysRevE.97.022611. 29548099

[ref78] Lotan I.; Head-Gordon T. An analytical electrostatic model for salt screened interactions between multiple proteins. J. Chem. Theory Comput. 2006, 2, 541–555. 10.1021/ct050263p. 26626662

[ref79] Yap E.-H.; Head-Gordon T. New and Efficient Poisson–Boltzmann Solver for Interaction of Multiple Proteins. J. Chem. Theory Comput. 2010, 6, 2214–2224. 10.1021/ct100145f. 20711494 PMC2920153

[ref80] Felberg L. E.; Brookes D. H.; Yap E.-H.; Jurrus E.; Baker N. A.; Head-Gordon T. PB-AM: An open-Source, fully analytical linear Poisson-Boltzmann solver. J. Comput. Chem. 2017, 38, 1275–1282. 10.1002/jcc.24528. 27804145 PMC5403608

[ref81] Yu Y.-K. Electrostatics of charged dielectric spheres with application to biological systems. II. A formalism bypassing Wigner rotation matrices. Phys. Rev. E 2019, 100, 012401 10.1103/PhysRevE.100.012401. 31499794

[ref82] Jackson J. D. Classical Electrodynamics, 3rd ed.; John Wiley & Sons Ltd.: Hoboken, NJ, 1999.

[ref83] Doerr T. P.; Obolensky O. I.; Yu Y.-K. Extending electrostatics of dielectric spheres to arbitrary charge distributions with applications to biosystems. Phys. Rev. E 2017, 96, 062414 10.1103/PhysRevE.96.062414. 29347333 PMC6312187

[ref84] Watson G. N. A Treatise on the Theory of Bessel Functions; Cambridge University Press: Cambridge, U.K., 1966.

[ref85] Hobson E. W. The Theory of Spherical and Ellipsoidal Harmonics; The University Press: Cambridge, U.K., 1931.

[ref86] Markovich T.; Andelman D.; Podgornik R. In Handbook of Lipid Membranes; Safinya C. R., Rädler J. O., Eds.; Taylor & Francis Group: Boca Raton, FL, 2021; Chapter 6, pp 99–128.

[ref87] Agra R.; Trizac E.; Bocquet L. The interplay between screening properties and colloid anisotropy: Towards a reliable pair potential for disc-like charged particles. Eur. Phys. J. E 2004, 15, 345–357. 10.1140/epje/i2004-10052-x. 15570447

[ref88] Quarteroni A. Numerical Models for Differential Problems, 3rd ed.; Springer: Cham, Switzerland, 2017.

[ref89] Siryk S. V. A note on the application of the Guermond-Pasquetti mass lumping correction technique for convection-diffusion problems. J. Comput. Phys. 2019, 376, 1273–1291. 10.1016/j.jcp.2018.10.016.

[ref90] Siryk S. V. Accuracy and stability of the Petrov-Galerkin method for solving the stationary convection-diffusion equation. Cybernetics and Systems Analysis 2014, 50, 278–287. 10.1007/s10559-014-9615-7.

[ref91] Sirik S. V. Estimation of the accuracy of finite-element Petrov-Galerkin method in integrating the one-dimensional stationary convection-diffusion-reaction Equation. Ukrainian Mathematical Journal 2015, 67, 1062–1090. 10.1007/s11253-015-1135-8.

[ref92] Gradshteyn I. S.; Ryzhik I. M. Table of Integrals, Series, and Products, 7th ed.; Academic Press, Elsevier: Boston, MA, 2007.

[ref93] Decherchi S.; Colmenares J.; Catalano C. E.; Spagnuolo M.; Alexov E.; Rocchia W. Between algorithm and model: different molecular surface definitions for the Poisson-Boltzmann based electrostatic characterization of biomolecules in solution. Commun. Comput. Phys. 2013, 13, 61–89. 10.4208/cicp.050711.111111s. 23519863 PMC3601494

[ref94] Horn R.; Johnson C. Matrix Analysis, 2nd ed.; Cambridge University Press: Cambridge, U.K., 2013.

[ref95] Lu S.; Pereverzyev S. V. Regularization Theory for Ill-Posed Problems: Selected Topics; De Gruyter: Berlin, Germany, 2013.

[ref96] Krasnoschok M.; Pereverzyev S.; Siryk S. V.; Vasylyeva N. Regularized reconstruction of the order in semilinear subdiffusion with memory. Springer Proceedings in Mathematics and Statistics 2020, 310, 205–236. 10.1007/978-981-15-1592-7_10.

[ref97] Krasnoschok M.; Pereverzyev S.; Siryk S. V.; Vasylyeva N. Determination of the fractional order in semilinear subdiffusion equations. Fractional Calculus and Applied Analysis 2020, 23, 694–722. 10.1515/fca-2020-0035.

[ref98] Salnikov N. N.; Siryk S. V. Parameter estimation algorithm of the linear regression with bounded noise in measurements of all variables. Journal of Automation and Information Sciences 2013, 45, 1–15. 10.1615/JAutomatInfScien.v45.i4.10.

[ref99] Filippov A. V.; Derbenev I. N. Effect of the size of charged spherical macroparticles on their electrostatic interaction in an equilibrium plasma. Journal of Experimental and Theoretical Physics 2016, 123, 1099–1109. 10.1134/S106377611611008X.

[ref100] Filippov A. V.; Derbenev I. N.; Pautov A. A.; Rodin M. M. Electrostatic interaction of macroparticles in a plasma in the strong screening regime. Journal of Experimental and Theoretical Physics 2017, 125, 518–529. 10.1134/S1063776117080040.

[ref101] Rocchia W. Poisson-Boltzmann equation boundary conditions for biological applications. Mathematical and Computer Modelling 2005, 41, 1109–1118. 10.1016/j.mcm.2005.05.006.

[ref102] Giordano S.; Rocchia W. Shape-dependent effects of dielectrically nonlinear inclusions in heterogeneous media. J. Appl. Phys. 2005, 98, 104101 10.1063/1.2128689.

[ref103] Matsuyama T.; Yamamoto H.; Washizu M. Potential distribution around a partially charged dielectric particle located near a conducting plane. J. Electrost. 1995, 36, 195–204. 10.1016/0304-3886(95)00048-8.

[ref104] Nakajima Y.; Sato T. Calculation of electrostatic force between two charged dielectric spheres by the re-expansion method. J. Electrost. 1999, 45, 213–226. 10.1016/S0304-3886(98)00051-5.

[ref105] Nakajima Y.; Matsuyama T. Electrostatic field and force calculation for a chain of identical dielectric spheres aligned parallel to uniformly applied electric field. J. Electrost. 2002, 55, 203–221. 10.1016/S0304-3886(01)00198-X.

[ref106] Washizu M. Precise calculation of dielectrophoretic force in arbitrary field. J. Electrost. 1993, 29, 177–188. 10.1016/0304-3886(93)90104-F.

[ref107] Washizu M.; Jones T. B. Multipolar dielectrophoretic force calculation. J. Electrost. 1994, 33, 187–198. 10.1016/0304-3886(94)90053-1.

[ref108] Washizu M.; Jones T. B. Dielectrophoretic interaction of two spherical particles calculated by equivalent multipole-moment method. IEEE Transactions on Industry Applications 1996, 32, 233–242. 10.1109/28.491470.

[ref109] Yu Y.-K. On a class of integrals of Legendre polynomials with complicated arguments – with applications in electrostatics and biomolecular modeling. Physica A: Statistical Mechanics and its Applications 2003, 326, 522–533. 10.1016/S0378-4371(03)00335-2. 15759366

[ref110] Dong S.-H.; Lemus R. The overlap integral of three associated Legendre polynomials. Applied Mathematics Letters 2002, 15, 541–546. 10.1016/S0893-9659(02)80004-0.

[ref111] Varshalovich D. A.; Moskalev A. N.; Khersonskii V. K. Quantum Theory of Angular Momentum; World Scientific: Singapore, 1988.

[ref112] Rashid M. A. Evaluation of integrals involving powers of (1 – *x*^2^) and two associated Legendre functions or Gegenbauer polynomials. Journal of Physics A: Mathematical and General 1986, 19, 2505–2512. 10.1088/0305-4470/19/13/016.

